# Distinct gene subsets in pterygia formation and recurrence: dissecting complex biological phenomenon using genome wide expression data

**DOI:** 10.1186/1755-8794-2-14

**Published:** 2009-03-10

**Authors:** Louis Tong, Jaime Chew, Henry Yang, Leonard PK Ang, Donald TH Tan, Roger W Beuerman

**Affiliations:** 1 Singapore Eye Research Institute (SERI), Singapore National Eye Center, Singapore; 2 Transcriptomics Platform, Singapore Immunology Network, Singapore; 3 Department of Cornea and External eye disease, Singapore National Eye Center, Singapore; 4 Department of Ophthalmology, Yong Loo Lin School of Medicine, National University of Singapore, Singapore

## Abstract

**Background:**

Pterygium is a common ocular surface disease characterized by fibrovascular invasion of the cornea and is sight-threatening due to astigmatism, tear film disturbance, or occlusion of the visual axis. However, the mechanisms for formation and post-surgical recurrence of pterygium are not understood, and a valid animal model does not exist. Here, we investigated the possible mechanisms of pterygium pathogenesis and recurrence.

**Methods:**

First we performed a genome wide expression analysis (human Affymetrix Genechip, >22000 genes) with principal component analysis and clustering techniques, and validated expression of key molecules with PCR. The controls for this study were the un-involved conjunctival tissue of the same eye obtained during the surgical resection of the lesions. Interesting molecules were further investigated with immunohistochemistry, Western blots, and comparison with tear proteins from pterygium patients.

**Results:**

Principal component analysis in pterygium indicated a signature of matrix-related structural proteins, including fibronectin-1 (both splice-forms), collagen-1A2, keratin-12 and small proline rich protein-1. Immunofluorescence showed strong expression of keratin-6A in all layers, especially the superficial layers, of pterygium epithelium, but absent in the control, with up-regulation and nuclear accumulation of the cell adhesion molecule CD24 in the pterygium epithelium. Western blot shows increased protein expression of beta-microseminoprotein, a protein up-regulated in human cutaneous squamous cell carcinoma. Gene products of 22 up-regulated genes in pterygium have also been found by us in human tears using nano-electrospray-liquid chromatography/mass spectrometry after pterygium surgery. Recurrent disease was associated with up-regulation of sialophorin, a negative regulator of cell adhesion, and *never in mitosis a*-5, known to be involved in cell motility.

**Conclusion:**

Aberrant wound healing is therefore a key process in this disease, and strategies in wound remodeling may be appropriate in halting pterygium or its recurrence. For patients demonstrating a profile of 'recurrence', it may be necessary to manage as a poorer prognostic case and perhaps, more adjunctive treatment after resection of the primary lesion.

## Background

Global gene expression has been used successfully to elicit biological behavior in different soft tissue tumors.[1] Pterygium as a human disease, noted to be more prevalent than 20% of some populations,[2,3] is of immense biological interest for a few reasons.

First, the pathogenesis of this condition is hotly debated. Hypothesis driven approaches have not resolved the relative importance of competing mechanisms for this disease. Theories that have been proposed include inflammatory influence,[4] degeneration of connective tissue,[5] genetic instability,[6] angiogenesis,[7] redox-related toxicity,[8] cellular proliferation,[9] aberration of apoptosis,[10] exuberant wound healing,[11] altered lipid metabolism,[12] mast cell infiltration.[13] and stem cell dysfunction.[5] Conventional approaches to disease mechanism, by virtue of their narrow focus, were not helpful to assess relative contribution of widely heterogenous processes. Furthermore, a fundamental issue about the diseased tissue remains un-resolved in this context: the origin of the epithelium overlying pterygial lesions, though suspected to be conjunctival in origin, is not entirely certain.[14]

Second, the spectrum of tumor size and behavior is tremendous, ranging from the inconspicuous lesion barely encroaching on the peripheral cornea, to the rapidly growing, menacing tumors that obscure the visual axes and threaten vision. In some contexts, additional molecular events.[15] may drive the original tumor to behave differently from the original lesion. It is also intriguing that unlike frank malignancies, these lesions, however fast growing, do not erode through the full thickness cornea, highlighting the presence of distinct processes from those manifested in malignant tumors.

Third, pterygium is a mixed soft tissue tumor that is strongly associated with non-ionising ultraviolet radiation.[4] Unlike the case of cutaneous melanomas, the cell type that responded to the environmental trigger may not be epithelial but rather, the fibro-vascular component[9] – a truly unusual phenomenon in human tumor biology because ultraviolet radiation-induced tumors encountered in humans are epithelial in origin and ultraviolet radiation effects on keloids, if any, are generally inhibitory.

Lastly, the inability of researchers to discover an analogous tumor in animals or reconstitute the disease in organ cultures imply that pterygium involves specie-specific mechanisms mediated by *in-vivo *cell-cell or cell-matrix interactions. The molecules involved in this disease may have therefore arisen from divergent evolution in the human ocular surface.

In view of the controversies on the multiple mechanisms of pterygia formation, we advocate an unbiased, global gene expression approach to decipher the dominant gene expression patterns that may underlie specific molecular events of interest. Recently, the genome-wide microarray data in whole tissue pterygium[16] as well as microarray data limited to cultured pterygial fibroblasts,[17] have been published. We have performed a study using gene microarray on recurrent pterygium, primary pterygium and un-involved conjunctiva, examined the gene subsets which were differentially regulated, compared our findings with these previous microarray studies,[16,17] and discussed some of the findings in the light of a complementary proteomics approach.[18]

In clinical practice, the treatment of this condition is surgical excision.[14] However, some cases aggressively recur after surgery.[14] Conjunctival auto-grafting as an adjunctive procedure may reduce recurrence, though the explanation for this is not entirely clear.[14] For these reasons, we speculate that a non-hypothesis driven approach may also be useful to discover gene expression signatures that predispose lesions to a more aggressive phenotype. Such information will benefit clinicians, who can appropriately anticipate otherwise 'un-expected' biological behavior in their treatment of this disorder.

## Methods

### Materials

Antibodies against MUC5AC, keratin 6 and CD24 were from Labvision, Neomarkers, Fremont, CA, USA, anti-MSMB antibodies were from US Biologicals, Massachusetts, MA, USA, anti-CEACAM5 and anti-NR4A2 antibodies were from Abcam, Cambridge, UK.

### Samples used for the Study

The procurement and use of both human tissues in this study was in compliance with the tenets of the Declaration of Helsinki. The study was approved by the Institutional Review Board of Singapore Eye Research Institute. Written informed consent was obtained from donors after explanation of the nature and possible consequences of the study. Human tissues samples were obtained from patients diagnosed with primary pterygium and came from different races, Chinese, Malay and Indian. All patients underwent pterygium excision in conjunction with the use of an upper bulbar conjunctival free autograft placed over the site of the original lesion. All pterygia specimens used were nasal pterygia. The whole pterygium tissue and a small portion of the conjunctival patch (approx. 1 × 3 mm) from the superotemporal conjunctiva were collected and were rapidly frozen in liquid nitrogen after removal and stored at -150°C. For immunohistology purpose, the samples were collected on ice and embedded in Optimal Cutting Temperature (OCT, Sakura, USA) in the laboratory.

### Microarray Experiment

The procedure for the microarray experiment has previously been published.[19] All microarray chips and related protocols and equipment for the processing of these chips were from Affymetrix Inc., Santa Clara, CA. The human genome GeneChip U133A consisting of more than 22000 probe sets was used for this study.

A group of 8 primary pterygium samples, harvested from 4 males (aged 40 to 50 years old) and 4 females (aged 50 to 60 years old) was used in this experiment. Another group of 4 control conjunctiva tissues, each pooled from 4 individual samples, was used as controls to the diseased tissues. Considering that only a tiny piece of the conjunctiva tissue could be obtained from each patient during the surgery, pooling of 4 conjunctiva tissues was necessary to obtain enough starting material.

Total RNA was extracted using TRIzol Reagent (Invitrogen, CA) and purified with RNeasy Mini Kit (Qiagen, Valencia, CA) according to the manufacturer's instructions. Five micrograms of each purified RNA sample were prepared according to the Affymetrix standard protocol. Fifteen micrograms of biotin-labelled cRNA using BioArray RNA Transcript Labelling Kit (ENZO Life Sciences, NY) were fragmented and the appropriate volume injected separately into the probe array chips.

The transcripts were hybridized onto the immobilized oligonucleotide sequence on array for 16 hours at 45°C under 60 rpm rotation using GeneChip Hybridization Oven 640. Washing and Strepavidin-staining steps were performed using Affymetrix Fluidics Station 450. The chips were scanned using GeneChip Scanner3000 and the image data were further analyzed using Microarray Suite v.5.0. Data analysis included pre-processing and normalization before identification of differentially expressed genes. Pre-processing adjusted for non-specific binding and background noise, whereas normalization removed systematic variation in the data due to effects other than biological differences. We used the Robust Multi-array Average (RMA) model [20] to extract the gene expression signals from probe intensities without taking the mismatch probe signals into consideration. Cross-array normalization was performed using the intensity-based log ratio median method [21] with the first array as the reference. Gene-level normalization was performed by normalizing all samples to the median of the expression level of the control (un-involved conjunctiva) samples. The data were annotated using gene annotation headings, bioprocesses and molecular functions from the NetsAffx database .

### Analysis of Microarray Data

The data reported in this study have been deposited in NCBI's Gene Expression Omnibus (GEO,  with GEO series accession number GSE2513). Data were visualised and explored using the GeneSpring GX 7.3 platform (Agilent Technology, Redwood City, CA).

For selection of differentially expressed genes, the modified *t*-statistic (SAM) [22] with 100% of the standard deviation percentile as the fudge constant was used. The threshold for significantly changed genes was set at a false discovery rate (FDR) of 5%. After delineating a list of differentially expressed genes, we performed various types of analysis to search for a pattern of global gene expression. The methods ranged from K-means clustering, to the construction of hierarchical dendrograms on a subset of genes, as well as the visualisation of gene expression pattern summarised by principal component analysis. In additional to analysis of differentially expressed genes, we employed the Gene Set Enrichment Analysis (GSEA) method for identification of those pathways that were more affected in pterygium tissues. The goal of GSEA is to determine whether members of a gene set tend to occur toward the top or bottom of the list, in which case the gene set is correlated with the phenotypic class distinction.[23] In contrast to the methods for extraction of differentially expressed genes, GSEA considers the collective up- or down-regulation of a gene set rather than individual genes. The GSEA software package downloaded from the Board Institute's website was used for the identification of activated or deactivated pathways in primary pterygial and conjunctival tissues. Microarray data for 8 primary pterygial samples and 4 samples of conjunctival tissue were used. Various datasets from publicly accessible databases (Biocarta, STKE, PubMed and KEGG) were dissected into over 300 gene sets. The two phenotypic classes used were pterygium and uninvolved conjunctiva tissues.

As an independent method of analyzing signaling pathways in pterygium, we exported the list of genes significantly up-regulated or down-regulated by 2 fold into the Pathway Studio 5.0 software (Ariadne Genomics Inc, Rockville, MD). The option 'Find all shortest paths between selected entities' was used on the up-regulated and down-regulated genes sequentially. The number of connectivities was limited to 2 and the genes mapped into known pathways displayed on a chart, and the relevant relationships listed in a table. The purpose of performing this analysis was to identify important upstream regulators of the differentially expressed genes as well as downstream effectors, in an unbiased fashion, based on known biological knowledge.

Methods used to extract and analyse tear proteins from patients who had undergone surgery for pterygium have already been described.[18] Wherever possible, the fold change data for genes corresponding to detected tear proteins were tabulated.

### Real Time Reverse Transcription Polymerase Chain Reaction

Six pairs of independent pterygium and uninvolved conjunctiva tissues were used in the real time reverse transcription polymerase chain reaction (qPCR) experiment. One microgram of the each total RNA preparation was reverse-transcribed to single stranded cDNAs using an oligo-dT primer with Superscript II Rnase H Reverse Transcriptase (Invitrogen, USA). Primer pairs specific to each gene used in the qPCR are listed in Additional file 1. qPCR was performed using SYBR Green PCR Master Mix (Applied Biosystems) in an ABI Prism 7700 Sequence Detection System (Applied Biosystems). The thermal cycling conditions were as follows: 95°C for 10 min, 45 cycles at 95°C for 30 s and 60°C for 1 min. Data obtained from qPCR were analyzed using the comparative CT method as previously described by Livak.[24] Paired t-statistics with *p*-value < 0.05 was used to determine whether the qPCR results in pterygial tissue were significantly different from those in un-involved conjunctiva.

### Immunohistochemistry

A separate group of paired pterygial and conjunctival tissue samples from three patients were used for immunohistochemistry. Sections were cut at 5 μm thicknesses from blocks of freshly frozen pterygium and matched un-involved conjunctiva embedded in the OCT. The post-fixed sections were incubated with the primary antibody at 4°C overnight. Specific antibodies against the following were used: MUC5AC, MSMB, CD24, CEACAM5, keratin 6 and NR4A2. After 3 washes with PBS, secondary antibody used was either fluorescein isothiocyanate (FITC)- or Rhodamine- conjugated anti IgG (Santa Cruz, USA), incubated with the sections for 40 minutes at room temperature. After 3 final washes each section was mounted with in a fluorescence mounting medium (DAKO, Denmark). Images were captured using a 40× Achrostigmat lens on an Axioplan2 microscope equipped with an AxioCam MR camera (Carl Zeiss, Germany).

## Results

### Global Gene Expression Profiling and analysis of pterygium versus conjunctiva

#### Gene expression analysis

**Figure 1:**
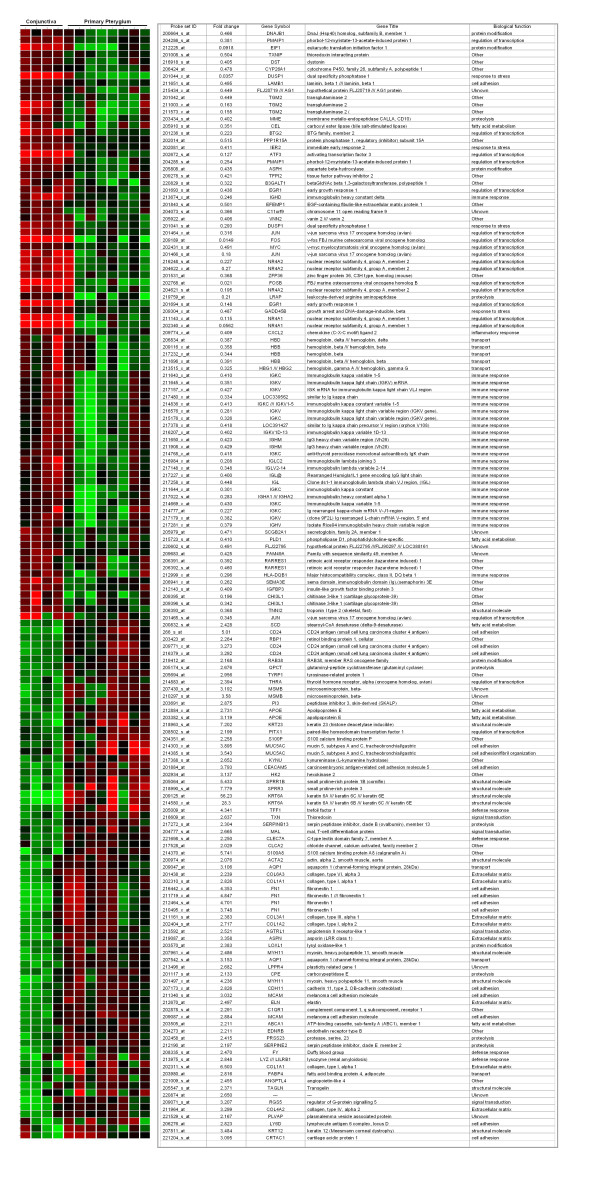
**Differentially expressed transcripts**. Heat map showing expression of 156 probe sets in conjunctiva and pterygium. Red represents high level of expression, green represents lower level expression and black represents no change.

With gene level normalization using un-involved conjunctival samples as reference, gene expression analysis showed that a total of 105 probe sets were significantly changed (p < 0.05) by at least 2 fold in primary pterygial tissue from uninvolved conjunctiva. Among these, 60 probe sets were up-regulated and 45 probe sets were down-regulated (Table 1). Without performing gene level normalization, 114 unique genes out of 156 probe sets were significantly changed in pterygium. (Figure 1)

**Table 1:** Genes significantly changed by at least 2-fold difference in pterygium (p < 0.05)

**Up-regulated in pterygium**			
**Probe ID**	**Gene** **symbol**	**Genbank accession**	**Fold change**

209125_at	KRT6A	J00269	6.399
205009_at	TFF1	NM_003225	5.671
210297_s_at	MSMB	U22178	4.175
211719_x_at	FN1	BC005858	4.112
212464_s_at	FN1	X02761	3.939
216442_x_at	FN1	AK026737	3.827
207811_at	KRT12	NM_000223	3.781
210495_x_at	FN1	AF130095	3.738
207430_s_at	MSMB	NM_002443	3.732
202310_s_at	COL1A1	K01228	3.635
216379_x_at		AK000168	3.475
209771_x_at	CD24	AA761181	3.42
218990_s_at	SPRR3	NM_005416	3.401
201884_at	CEACAM5	NM_004363	3.287
214385_s_at	MUC5B	AI521646	3.244
202404_s_at	COL1A2	NM_000089	3.149
205064_at	SPRR1B	NM_003125	3.053
204777_s_at	MAL	NM_002371	3.043
211161_s_at		AF130082	2.937
219087_at	ASPN	NM_017680	2.903
214303_x_at	MUC5AC	AW192795	2.897
209047_at	AQP1	AL518391	2.773
213796_at	SPRR1A	AI923984	2.772
205694_at	TYRP1	NM_000550	2.736
214580_x_at	KRT6A	AL569511	2.645
217272_s_at	SERPINB13	AJ001698	2.545
218963_s_at	KRT23	NM_015515	2.506
218353_at	RGS5	NM_025226	2.476
205547_s_at	TAGLN	NM_003186	2.467
217388_s_at	KYNU	D55639	2.441
201438_at	COL6A3	NM_004369	2.423
203477_at	COL15A1	NM_001855	2.374
203980_at	FABP4	NM_001442	2.364
202311_s_at	COL1A1	AI743621	2.334
215076_s_at	COL3A1	AU144167	2.331
213975_s_at	LYZ	AV711904	2.322
209071_s_at	RGS5	AF159570	2.275
210809_s_at	POSTN	D13665	2.261
202878_s_at	C1QR1	NM_012072	2.242
217528_at	CLCA2	BF003134	2.226
204682_at	LTBP2	NM_000428	2.21
212190_at	SERPINE2	AL541302	2.201
219410_at	FLJ10134	NM_018004	2.171
209848_s_at	SILV	U01874	2.15
204351_at	S100P	NM_005980	2.114
204971_at	CSTA	NM_005213	2.092
206427_s_at	MLANA	U06654	2.088
202917_s_at	S100A8	NM_002964	2.084
203382_s_at	APOE	NM_000041	2.081
211980_at	COL4A1	AI922605	2.077
200665_s_at	SPARC	NM_003118	2.071
201667_at	GJA1	NM_000165	2.06
218723_s_at	RGC32	NM_014059	2.056
203691_at	PI3	NM_002638	2.05
206276_at	LY6D	NM_003695	2.04
219412_at	RAB38	NM_022337	2.04
208982_at	PECAM1	AW574504	2.029
203504_s_at	ABCA1	NM_005502	2.026
201852_x_at	COL3A1	AI813758	2.01
203570_at	LOXL1	NM_005576	2.01

**Down-regulated in pterygium**			
**Probe ID**	**Gene**	**Genbank accession**	**Fold change**

217232_x_at	HBB	AF059180	0.5
208078_s_at	TCF8	NM_030751	0.499
201842_s_at	EFEMP1	AI826799	0.496
201008_s_at	TXNIP	AA812232	0.495
215176_x_at		AW404894	0.494
206391_at	RARRES1	NM_002888	0.494
212225_at	SUI1	AL516854	0.492
201505_at	LAMB1	NM_002291	0.488
205979_at	SCGB2A1	NM_002407	0.487
209278_s_at	TFPI2	L27624	0.482
211003_x_at	TGM2	BC003551	0.479
211573_x_at	TGM2	M98478	0.479
202431_s_at	MYC	NM_002467	0.475
213831_at	HLA-DQA1	X00452	0.47
210095_s_at	IGFBP3	M31159	0.465
206392_s_at	RARRES1	NM_002888	0.46
211651_s_at	LAMB1	M20206	0.455
201843_s_at	EFEMP1	NM_004105	0.443
202081_at	IER2	NM_004907	0.439
202340_x_at	NR4A1	NM_002135	0.435
200664_s_at	DNAJB1	BG537255	0.435
209116_x_at	HBB	M25079	0.431
215723_s_at	PLD1	AJ276230	0.43
205910_s_at	CEL	NM_001807	0.417
219759_at	LRAP	NM_022350	0.416
204286_s_at	PMAIP1	NM_021127	0.406
206424_at	CYP26A1	NM_000783	0.403
206393_at	TNNI2	NM_003282	0.396
201466_s_at	JUN	NM_002228	0.388
201464_x_at	JUN	BG491844	0.368
212143_s_at	IGFBP3	BF340228	0.359
201473_at	JUNB	NM_002229	0.356
217022_s_at	MGC27165	S55735	0.355
201236_s_at	BTG2	NM_006763	0.342
204285_s_at	PMAIP1	AI857639	0.301
201041_s_at	DUSP1	NM_004417	0.296
201044_x_at	DUSP1	AA530892	0.265
204622_x_at	NR4A2	NM_006186	0.22
201694_s_at	EGR1	NM_001964	0.215
204621_s_at	NR4A2	AI935096	0.192
213674_x_at	IGHM	AI858004	0.188
216248_s_at	NR4A2	S77154	0.17
202768_at	FOSB	NM_006732	0.167
202672_s_at	ATF3	NM_001674	0.159
209189_at	FOS	BC004490	0.072

#### Gene Ontology Analysis

**Figure 2:**
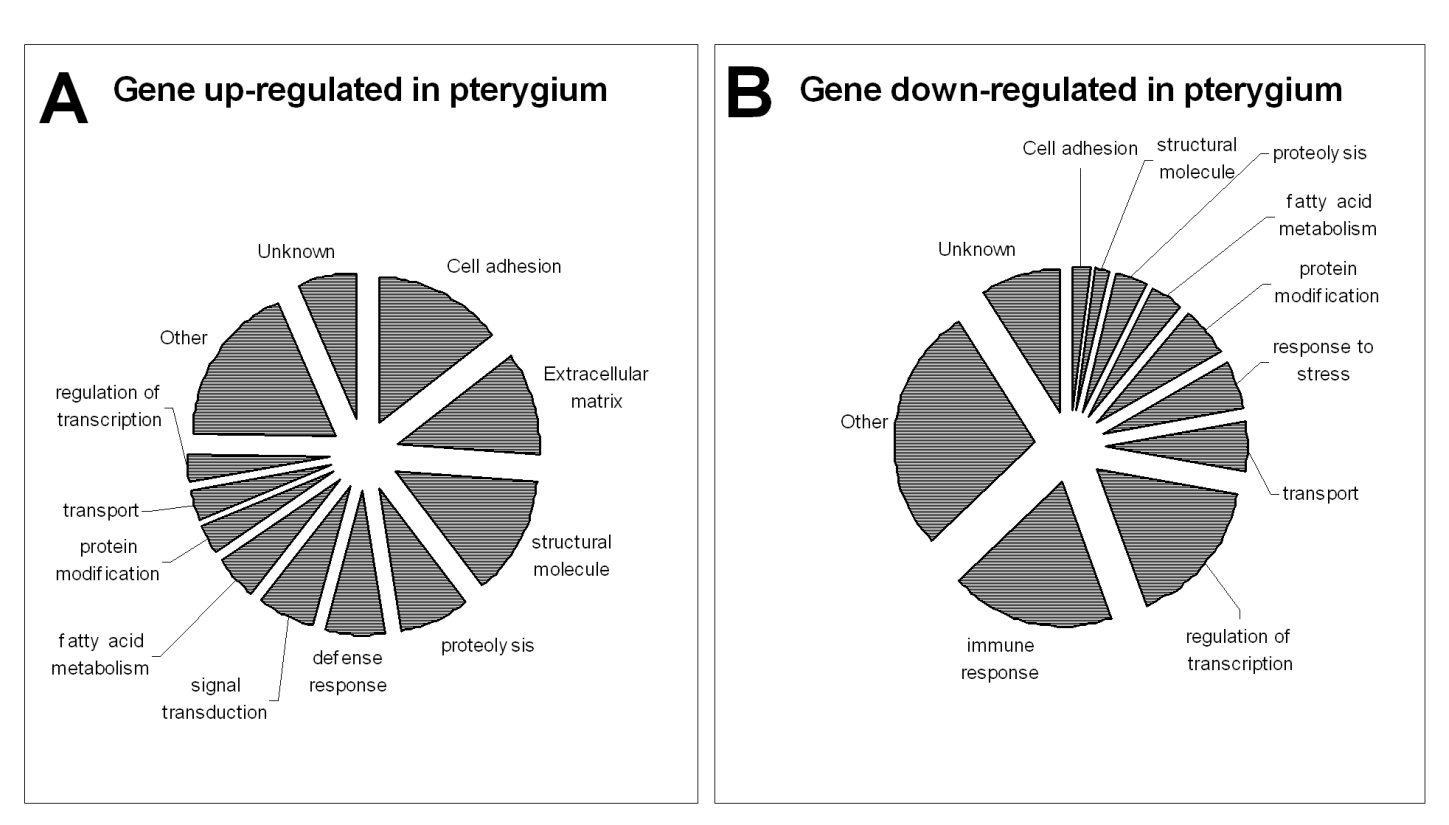
**Biological function of dysfunctional genes**. Pie Charts showing the percentage of genes with different biological functions that were dysregulated in pterygium.

All significantly changed genes were categorized based on biological functions using categories from the GeneOntology database (Figure 2). Among the up-regulated genes, genes coding for cell adhesion (8), extracellular matrix (ECM) (7) and structural proteins (8) accounted for 38% of up-regulated genes. Examples of up-regulated cell adhesion molecules include CADH11, MCAM, FN1, CEACAM5, ECM component include COL1A2, COL1A1, COL4A2, COL6A3 and structural proteins, ACTA2, KRT12, KRT6, KRT23, SPRR1B and SPRR3. Several of these genes also encode for proteins involved in wound healing, including COL6A3, CD24, FN1, KRT6a, TFF1, SPRR1b, and MUC5AC. Another group of up-regulated genes is the 'mitogenic proteins' group exemplified by MSMB, CEACAM5 and CD24.

Some immune response and transcription factors were down-regulated in primary pterygium. Examples of the former include immunoglobulin heavy chain genes, whereas examples of the latter include ATF3, BTG2, ERG1, FOS, FOSB, JUN, NR4A1 and NR4A2. Other down-regulated genes include those encoding for transport proteins, *ie*., HBB and HBD, and those involved in stress-response, including DUSP1 and GADD45B.

When the composition of the list of significantly changed genes was studied in terms of certain gene ontology categories, we observed that there is an over-representation of such genes compared to their expected frequency, considering the proportion of the probes representing these genes present on the chip (Table 2). This suggests that structural molecules and molecules with transporter activity play a role in pterygium.

**Table 2:** Proportion of significantly changed genes by functional category.

**Gene Ontology (GO)**	**Observed %**	**Expected %**	**p value**
Extracellular region (5576)	16.3	5.3	0.00698
Extracellular matrix (31012)	13.95	2.4	0.000503
Intermediate filament cytoskeleton (45111)	6.977	0.53	0.00197
Intermediate filament (5882)	6.977	0.58	0.00197
Muscle development (7517)	6.977	1.16	0.0135
Transporter activity (5215)	23.26	12.06	0.0291
Structural molecule activity (5198)	20.93	5.424	0.000414
Extracellular matrix structural constituents (5210)	9.302	0.675	0.000198
Oxidoreductase activity acting on paired donors (16705)	6.977	0.631	0.0025

Note: This refers to primary pterygium versus control. Only functional categories which represent at least 5% of the significantly changed genes are shown. The numbers in the brackets represent the GO category ID.

#### Analysis of enriched pathways

**Figure 3:**
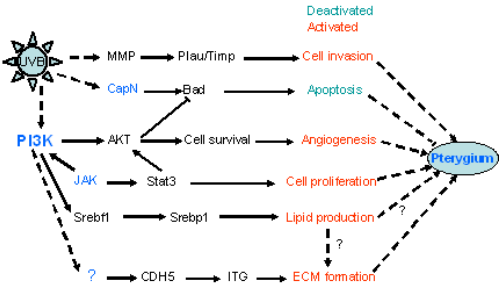
**Gene Set Expression analysis**. Diagram showing the affected pathways involved in pathogenesis of pterygium by Gene Set Expression analysis.

Gene set enrichment analysis (GSEA) was performed using gene expression data from the conjunctival and primary pterygium groups (Figure 3). Significantly enriched pathways in pterygium were related to ECM formation, tissue invasion, angiogenesis and lipid metabolism. Candidate genes found to be involved in the ECM pathway were cadherins (CDH5 and CDH3), integrins (eg., ITGb1), cystatin C (CST3) and intracellular adhesion molecule 1(ICAM1). Angiogenesis related genes include akt1, akt2, tyrosine kinase 2 (Tyk2), phosphinositide-3-kinases (PIK3C2A, PIK3CD, PIK3R1, PIK3R2, PIK3R4) and janus kinases (JAK1, JAK2). The genes involved in 'tissue invasion' pathways include plasminogen activator and receptor (PLAU, PLAUR), matrix metalloproteinase (MMP1,2,9), tissue inhibitor of metalloproteinase 1 (TIMP1, TIMP3), serine (or cysteine) proteinase inhibitor clade B member 5 (SERPINB5), s100 calcium binding protein (S100A4), secreted phosphoprotein1 (spp1) and mucin (MUC1). The lipid formation pathway was also significantly enriched in pterygium tissues, for example, the sterol regulatory element binding transcription factor1 (SREBF1), dual specific phosphatase 14 (DUSP14), hexokinase2 (HK2) and the solute carrier family 2 member 4 (SLC2A4).

The only significantly enriched pathway in un-involved conjunctiva was related to apoptosis. The genes in this pathway include the calpains (CAPN1, CAPN2, CAPN3, CAPNS1) and bcl-antagonist of cell death (BAD2).

#### Pathway analysis to identify upstream and downstream signals

**Figure 4:**
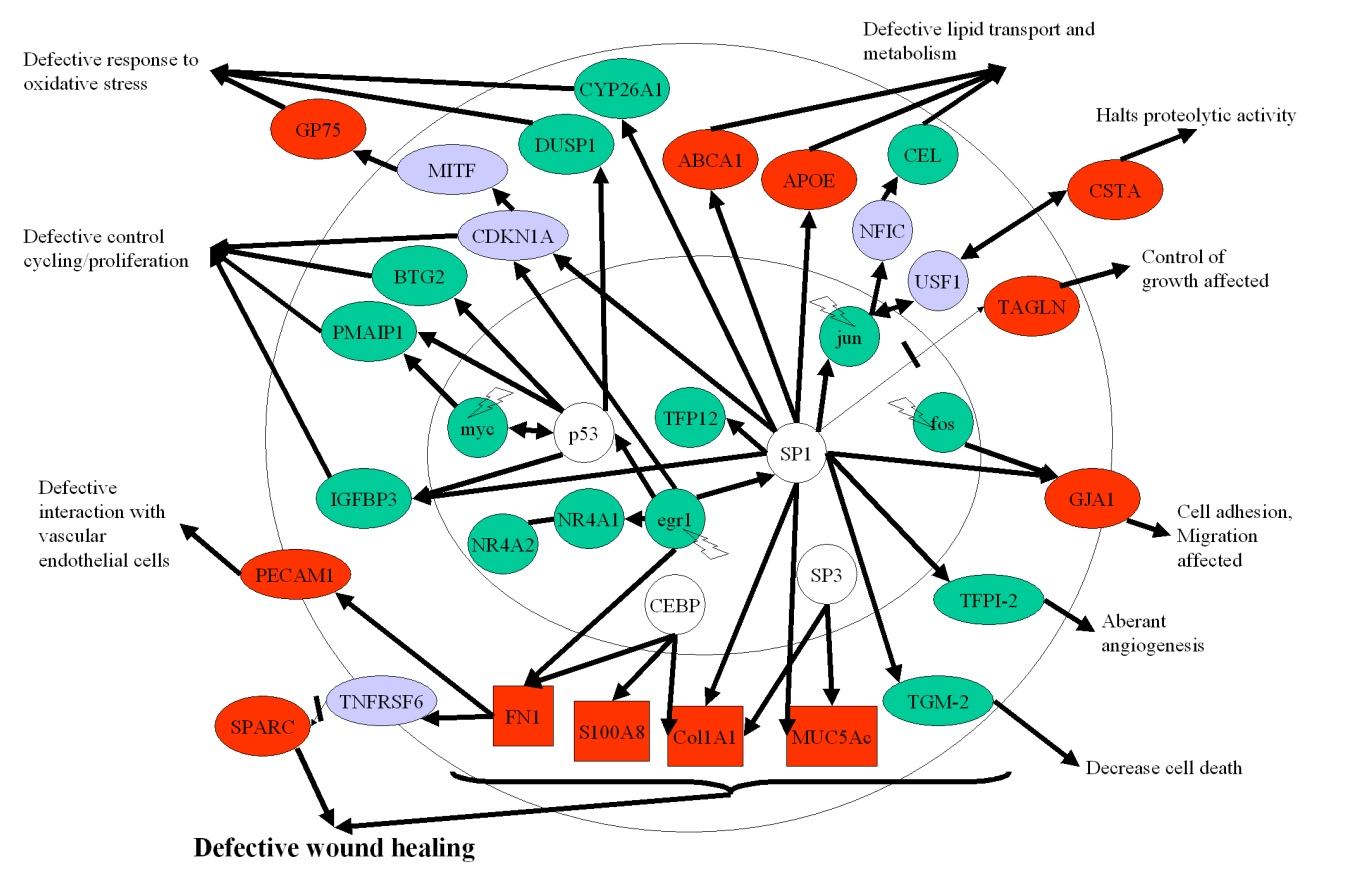
**Signaling in pterygium**. Schematic representation of potential signaling pathways involved in primary pterygium. These pathways could be affected in conjunctival epithelial cells, fibroblasts or vascular endothelial cells. Pathways were identified by incorporating the microarray results (genes which were differentially expressed between normal conjunctival and pterygium tissue) into Pathway Studio. Green symbols represent down-regulation, whereas red symbols represent up-regulation of genes. Solid lines represent positive regulation, thin arrow with a cross line represent inhibition. Purple symbols represent intermediate molecules in the potential pathways that may have altered function, for example, CDKN1A or p21 (Cip) may have reduced function due to down-regulated egr1 transcription factor. GP75: Tyrosinase-related protein 1, CSTA: cystatin A, TAGLN: transgelin, GJA1: Gap junction protein α-1 or Connexin-43, ABCA1: ATP binding cassette subfamily A1 protein, CEL: carboxyl ester lipase, DUSP1: dual specificity phosphatase I, PMAIP1: phorbol-12-myristate-13-acetate-induced protein 1, BTG2: B cell translocation gene 2, CYP26A1: cytochrome P450 family 26 A1 isoform, NR4A1 and 4A2: nuclear receptors 4A1 and 2, SPARC: secreted protein acidic rich in cysteine, TGM-2: transglutaminase 2, TFPI-2: Tissue factor pathway inhibitor 2 and IGFBP3: insulin-like growth factor binding protein 3. Green symbols within the nucleus represent transcription factors genes that were depressed, probably due to the upstream stress signaling such as those related to ultraviolet light (not shown). Due to quenching effect, there may be an increased in the transcriptional promoter activity of other transcription factors such as SP1, CEBP and SP3 (open symbols).

Up-regulated genes and down-regulated genes were analysed in pathway studio. Figure 4 summarised signaling network that resulted from this analysis. Primary pterygium may be characterized by stress-induced down-regulation of transcription factors (Egr1, Jun and Fos), with defective wound healing as the major process responsible for the disease phenotype. Scrutiny of the networks in Figure 4 illustrates a few other relevant processes: up-regulation of PECAM1 and down-regulation of TFPI-2 may contribute to aberrant vascularisation, whereas down-regulation of DUSP1 and up-regulation of GP75 may contribute to an abnormal response to oxidative stress. The identities and biological/molecular functions of these relationships are shown in Additional files 2 and 3.

#### Clustering analysis

To extract some useful knowledge or patterns from global gene expression data, we performed a few cluster analyses. The K-means cluster method was computed using 5 clusters on the 156 gene probes differentially expressed in pterygium compared to controls, over 1000 iterations. Table 3 shows the final characteristics of the clusters. Two clusters contained genes that were down-regulated in pterygium, whereas three other clusters showed genes that were up-regulated. Visual examination of the role of these genes revealed biological functions that are consistent with the above-mentioned pathway and gene ontology studies.

**Table 3:** K-means cluster analysis supplemented with GO browser results.

**Cluster**	**No of Genes**	**Average Radius**	**Status in pterygium**	**Major function by GO**
1	58	2.463	Down-regulated	Response to stimulus
2	13	1.803	Up-regulated	Metabolism
3	37	1.507	Up-regulated	Cell adhesion, response to wounding
4	22	2.357	Up-regulated	Lipid related
5	26	1.827	Down-regulated	Cell proliferation

**Figure 5:**
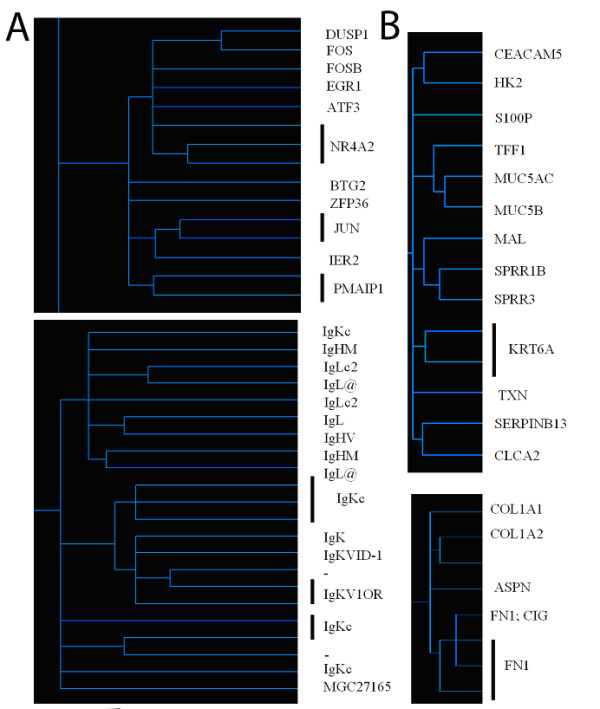
**Hierarchical clustering analysis**. Dendrograms were constructed using hierarchical clustering algorithms, performed on the subset of significantly changed genes. The shorter the length of the branches, the more co-expressed the members of the genes are. Magnified portions of the dendrograms showing the clusters of co-expression for genes down-regulated (A) and up-regulated (B) in primary pterygium relative to control. Note that the 2 clusters in A involved transcription factors and immunoglobulins, whereas 2 clusters in B were involved in structural proteins and extracellular matrix.

To identify co-expressed gene sets or the similarity of samples, we further performed hierarchical clustering analysis on the data for the 156 gene probes. Two closely related clusters of genes up-regulated in conjunctiva was detected (Figure 5A), and similarly, 2 clusters of up-regulated genes in pterygium were detected (Figure 5B). Scrutiny of the composition of these clusters yields a number of cytoskeletal proteins, immunoglobulins, cancer markers and transcription factors.

**Figure 6:**
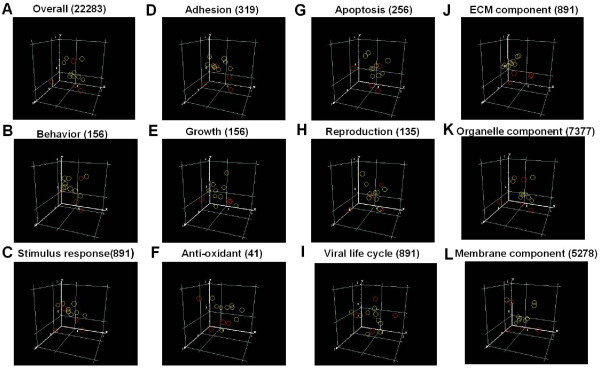
**Principal component analysis**. Three-D scatter plots constructed from principal component analyses of gene expression data. In the scatter plots the first, second and third principal components were plotted on the x, y and z axis respectively. Red circles represent conjunctival samples (control) whereas yellow circles denote primary pterygial samples. A. Performed on all genes, B-L. Using subsets of Gene Ontology genes corresponding to a biological process or molecular function and J-L. Using subsets of Gene Ontology genes corresponding to specific cellular components. Brackets represent the number of genes in the overall analysis (A) or in subsets (B-L)

We further performed principal component analysis on the entire set of genes in the chip, as well as several selected categories of Gene Ontology previously implicated in the pathogenesis of pterygium (Figure 6) for the following two purposes: 1. To show the compactness of the samples (represented by the distance between circles) in each condition and 2. To reveal the possible existence of a plane which can separate the 2 conditions. The 3D scatter plots of the first three prinicipal components show that the expression of genes coding for ECM component (Figure 6J) was able to differentiate the conjunctiva and pterygium samples better than those in the antioxidant (Figure 6F) and apoptosis (Figure 6G) categories. When all the genes were included in the analysis (Figure 6A), a clear plane between the pterygial and conjunctival samples was not so evident. This may be due to the inclusion of many genes that were not significantly regulated but whose expression data contained extensive noise. For this analysis, we deliberately included processes like 'reproduction' (Figure 6H) being unlikely to play a significant role in pterygium pathogenesis, acted as a negative control.

#### Differences between primary and recurrent pterygium

Recurrent pterygium [see Additional file 4] differs from primary cases as it has a different morphology and often worse prognosis. Table 4 shows the genes that were differentially regulated in recurrent compared to primary pterygium. Additional file 5 shows that a large number of genes in recurrent pterygium were down-regulated or up-regulated relative to primary pterygium and conjunctiva tissues. Since there were a rather large number of differentially regulated genes, only those with at least 2 fold change between primary and recurrent pterygia are shown in table 4. It is interesting that the up-regulated genes are not the same as those up-regulated in primary pterygium compared to conjunctiva. Examples include stearoyl-CoA desaturase 5 that converts fatty acids into monounsaturated forms in the endoplasmic reticulum and involved in dyslipidemia, the ubiquinol-cytochrome c reductase involved in the mitochondrial respiratory chain, the DNA recombination repair protein RAD51, the neuronal thread protein (AD7C-NTP), which is a extracellular protein involved in apoptosis, and the gene for Mediterranean fever (MEFV) which is involved in regulation of transcription and inflammatory response. Involvement of the gene for sialophorin (SPN or CD43) and NEK5 (Never in mitosis gene a) are consistent with increased cell migration because SPN is a negative regulator of cell adhesion and NEK5 is involved in microtubule function and motility. Additional file 6 shows possible mechanisms of pterygium recurrence involving these mediators.

**Table 4:** Distinguishing recurrent from primary pterygia (p < 0.05), list of genes down-regulated or up-regulated by at least 2-fold.

**A. Name of down-regulated genes**	**No of folds down-regulated**	**B. Name of up-regulated genes**	**No of folds up-regulated**
PSCA	-4.34	SCD5	4.05
COL1A1	-4.21	C12orf38	3.12
COL6A2	-3.24	MEFV	3.10
COL3A1	-3.09	UQCRQ	2.68
HDDC2	-2.84	RAD51L3	2.43
ASPN	-2.77	SPN	2.36
YWHAE	-2.66	AD7C-NTP	2.19
KRT7	-2.65	NEK5	2.18
THRAP5	-2.62	LOC389286	2.00
HNRPH2	-2.60		
UBQLN2	-2.43		
FLJ20273	-2.38		
MUC7	-2.38		
UNC84A	-2.35		
MSMB	-2.34		
LTBP3	-2.33		
NDFIP1	-2.31		
BAT1	-2.30		
AKAP9	-2.28		
SMC1A	-2.24		
BNIP3L	-2.23		
COPE	-2.22		
CAPZA2	-2.20		
RBM3	-2.18		
PKM2	-2.18		
SLC34A2	-2.15		
C9orf95	-2.15		
ZDHHC11	-2.14		
SPARC	-2.12		
DICER1	-2.07		
LOXL1	-2.07		
SPINT1	-2.06		
LSM14A	-2.06		
PSMC6	-2.06		
UBE1	-2.06		
MARCKS	-2.05		
PFN1	-2.04		
H1FX	-2.03		
DDX41	-2.03		
MGP	-2.02		
NDUFS8	-2.01		
PSCA	-4.34		

### Validation of gene microarray data

**Figure 7:**
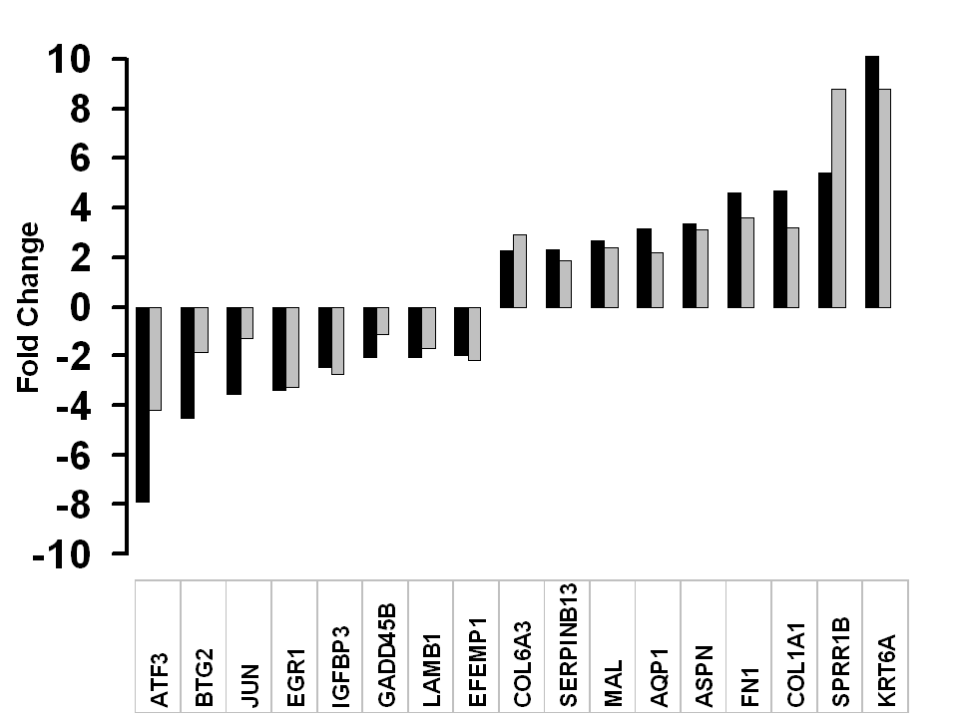
**Validation of microarray data**. Bar graph showing the correlation of microarray data with real time PCR transcript levels. Black bars represent number of folds of change by GeneChip experiment, gray bars represent the number of folds of change using real time PCR. A normalised ratio (Y-axis) of more than 1 indicates up-regulation in pterygium, whereas a ratio of less than 1 indicates down-regulation in pterygium. The X axis shows an arbitrarily selected panel of genes.

Relative quantitative real time polymerase chain reaction (qPCR) was performed as an independent laboratory approach to validate the transcriptional level changes in the microarray experiment. These results (Figure 7) show that for all genes selected for validation with qPCR, the direction and magnitude of changes were consistent with the results obtained from the microarray analysis.

**Figure 8:**
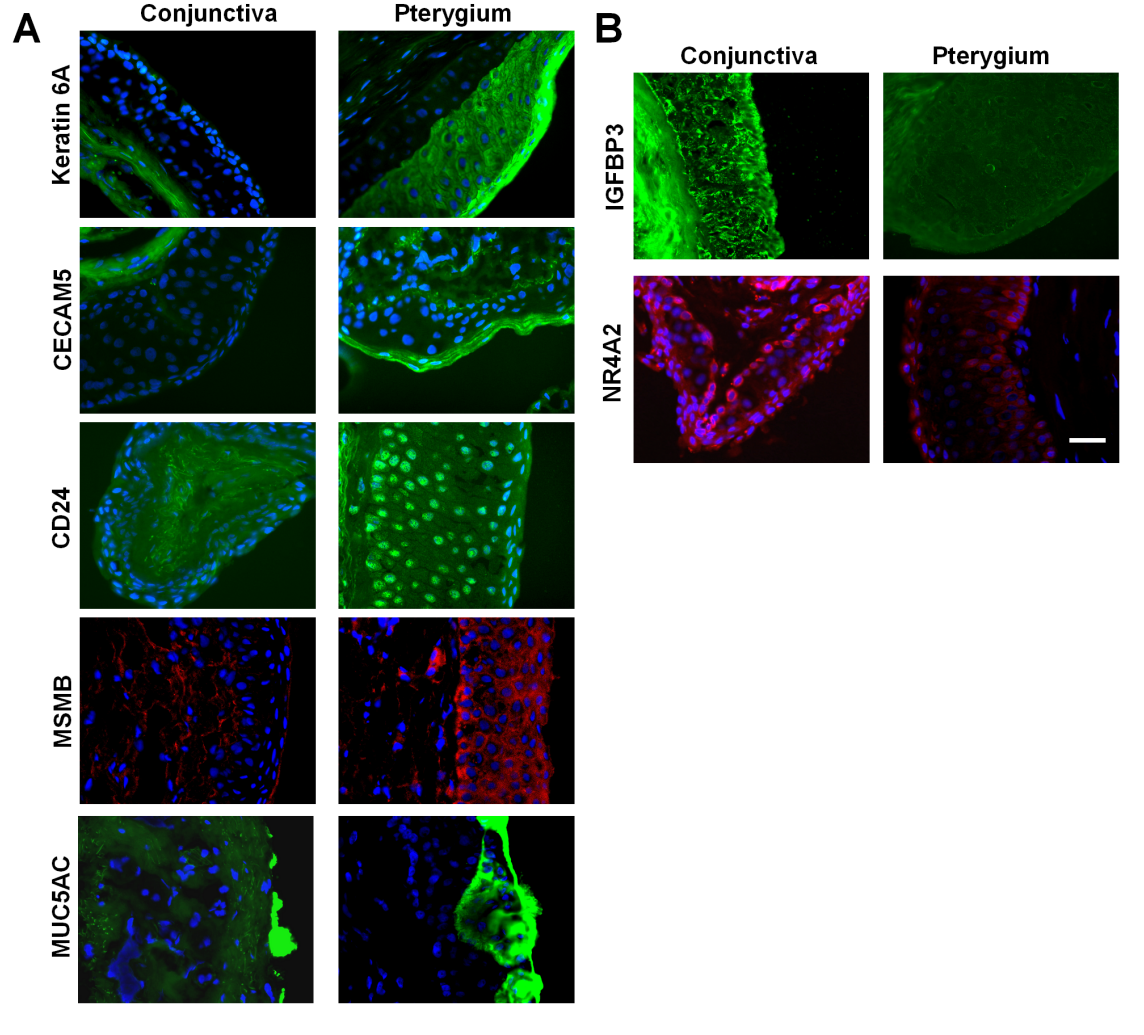
**Tissue localisation of important molecules in pterygium**. Immunohistochemical staining images of uninvolved conjunctiva and pterygium tissue. Nuclear position (and indirectly, tissue architecture) was shown using counter-staining with DAPI, in blue. The same magnification was used in all images: Scale Bar = 100 micrometers. A. Proteins with increased expression in pterygium. Staining for keratin 6A, CECAM5, CD24 and MUC5AC was shown in green and MSMB, in red. B. Proteins with decreased expression in pterygium. Staining for IGFBP3 and NR4A2 was shown in green and red respectively.

We then addressed the question whether protein levels were also affected in pterygium. Several proteins were chosen arbitrarily to address the protein expression levels as well as the localization in pterygium tissues. Our results (Figure 8) show that protein expression levels for keratin 6, CEACAM5, CD24, MSMB and MUC5AC were increased, whereas NR4A2 and IGFBP3 were reduced in pterygium, consistent with the transcript changes detected by the microarray analysis. Furthermore, the immunofluorescent staining yielded interesting information about the localization of these proteins in pterygium. Immunofluorescent staining (Figure 8A) shows that keratin 6a stained strongly in the superficial layer in pterygial epithelium but not in un-involved conjunctiva. Similarly, CEACAM5 was detected in the squamous layer of pterygial epithelium but was not detectable in conjunctiva. There was up-regulation and nuclear accummulation of the cell adhesion molecule CD24 in the pterygium epithelium, but CD24 was not detectable in conjunctiva. MSMB, on the other hand, was detected prominently in the basal epithelial layer of pterygium, with some staining also in the superficial stromal cells adjacent to the basal epithelia. Lastly, MUC5AC protein was detected in both pterygial and conjunctival tissue sections predominantly in goblet cells. However, the intensity of MUC5AC staining in pterygium was stronger than that in conjunctiva. IGFBP3, present in un-involved conjunctival stroma and epithelium, and NR4A2, present in conjunctival epithelium, were reduced in pterygium (Figure 8B).

**Figure 9:**
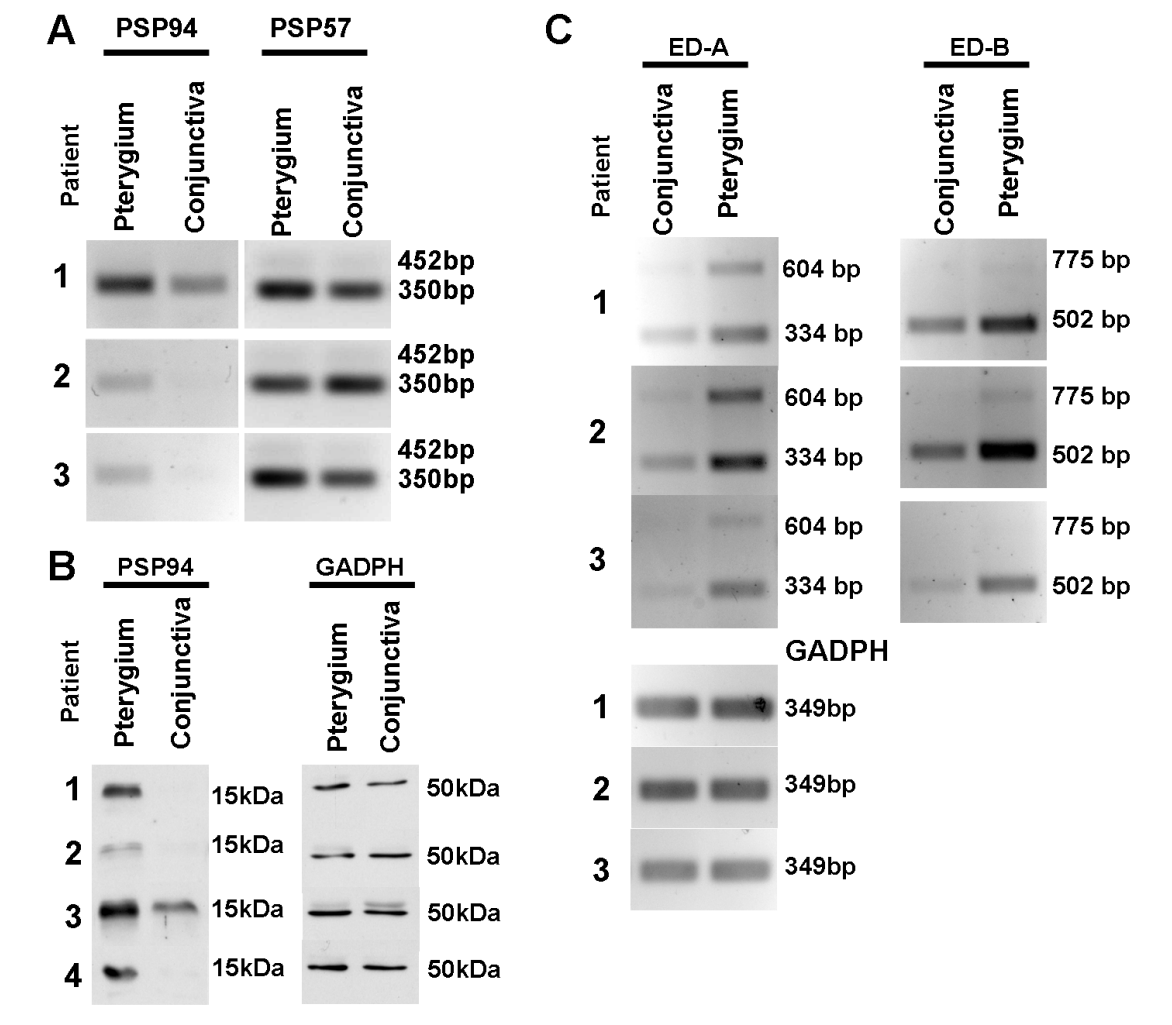
**Important molecules in pterygium pathogenesis**. A and C. Ethidium bromide stained gel images from semi-quantitative reverse transcription polymerase chain reaction. A. Using specific primers against PSP57 (amplicon length of 452 bp) and PSP94 (amplicon length of 350), PCR products corresponding to PSP94 could be visualised at 30 cycles, whereas PSP57 products could only be just detected at 40 cycles. The sequences were confirmed by sequencing the specific band after cutting the band and extraction of DNA. Note that in patients 1–3, PSP94 transcripts were up-regulated in pterygium relative to conjunctival controls. B. Western blot images, using specific antibodies against MSMB and GADPH (loading control). Note that the PSP proteins were up-regulated in pterygium relative to controls. C. The alternative spliced forms of fibronectin transcripts ED-A and ED-B were detected by visualisation of PCR products. Note that all forms of the transcripts were up-regulated in pterygium compared to conjunctival controls.

The up-regulation of MSMB transcripts (Figure 9A) and proteins (Figure 9B) was also verified by semi-quantitative reverse transcription polymerase chain reaction and Western blot respectively.

### Comparison of microarray data with tear proteins

Another report from our center has previously documented the analysis of tear proteins in tears of patients after surgery for pterygium.[18] Briefly, this study employed reverse-phase high-pressure liquid chromatography followed by tryptic digestion and characterisation of proteins using nanoLC-nano-ESI-MS/MS. Eleven genes corresponding to the detected tear proteins were significantly up-regulated in pterygium compared to un-involved conjunctival tissue (Table 5). Examples of the up-regulated genes were those encoding for prolactin induced protein I and the S100 A8 proteins. Another eleven genes corresponding to the detected tear proteins were found to be down-regulated in pterygium. These include lipophilin C, ribonuclease 4, complement C3 and histone 1b. Twenty-nine genes corresponding to detected tear proteins were not significantly up or down-regulated in pterygium. Since there were no controls in this study,[18] it is difficult to interpret some of these findings. Furthermore it was not possible to compare the microarray results with every instance of the protein data because more than one type of probe sets may contribute to the synthesis of the protein and not all gene probe sets have signal-noise ratio high enough for this analysis.

**Table 5:** Gene microarray data corresponding to detected tear proteins.

**Name of gene corresponding to tear protein**	**Genbank Accession**	**No of fold change**	**p value**
**A. Genes significantly regulated in pterygium**			
Prolactin-induced protein I	NM_002652	2.149	0.000213
S100A8 (Calgranulin A)	NM_002964	1.74	0.0000082
Defensin alpha A1	U73945	1.506	0.0149
S100 A9	NM_002965	1.442	0.00204
Hepatocellular carcinoma associated protein (PIGR)	NM_002644	1.371	0.0299
Vimentin	AI922599	1.278	0.0324
Beta-2 microglobulin	NM_004048	1.188	0.0378
Cathepsin G	NM_001911	1.141	0.00984
S100 A12	NM_005621	1.122	0.0268
Clusterin (Apolipoprotein J)	AI982754	1.08	0.0287
Vitamin D binding protein	NM_000583	1.052	0.0437
Cystatin SN	NM_001898	0.946	0.0121
Histone 1b	NM_005322	0.941	0.00456
Orosomucoid 1 (alpha 1 acid glycoprotein)	NM_000607	0.94	0.0027
Anti-thrombin III	D29832	0.937	0.000451
Beta actin	X00351	0.888	0.0263
Proline rich Peptide	NM_025263	0.864	0.00355
Ribonuclease 4	NM_002937	0.725	0.000675
Alpha-1-antitrypsin (SERPINA1)	NM_000295	0.72	0.0399
Ceruloplasmin (ferroxidase)	NM_000096	0.637	0.000375
Complement C3	NM_000064	0.607	0.00443
Lipophilin C (SCGB2A1)	NM_002407	0.402	0.000045
**B. Genes not significantly regulated in pterygium**			
IGHM protein	L23517	0.89	0.0511
Lipophilin A	NM_006552	0.955	0.073
Zn-alpha2-glycoprotein	D90427	1.223	0.0788
Apolipoprotein A-I	NM_000039	0.934	0.0874
Immunoglobulin kappa light	M63438	0.559	0.0878
Mucin 7	L13283	1.023	0.0895
Lipocalin 1	NM_002297	0.949	0.135
Alpha-fibrinogen	NM_021871	1.038	0.261
Transferrin	A1073407	0.97	0.319
Proline rich 4 (lacrimal)	NM_007244	0.959	0.32
Nasopharyngeal carcinoma associated proline rich protein 4	NM_007244	0.959	0.32
Lactoferrin	NM_002343	1.068	0.366
Complement Factor-B	AF349679	1.048	0.386
Immunoglobulin J chain	AV733266	0.707	0.398
cystatin SA	NM_001322	0.977	0.415
Apolipoprotein A-IV	NM_000482	0.977	0.442
Transthyretin	AH62690	1.024	0.493
Lysozyme	NM_020426	0.982	0.527
Complement factor H	X04697	0.956	0.542
Annexin I	NM_000700	0.959	0.583
Secretary leukocyte protease inhibitor	NM_003064	0.944	0.592
Proline rich protein 5	NM_012390	1.145	0.597
Coagulation factor H (thrombin)	NM_000506	0.979	0.6
Neutrophil elastase	NM_001972	0.99	0.68
Apolipoprotein H	NM_000042	1.013	0.691
Hemopexin	BC005395	1.013	0.7
Albumin	AF116645	1.007	0.8
Haptoglobin	NM_005143	1.005	0.836
Basic proline rich protein	X07882	1.004	0.905

Note: the study did not have expression data for the genes corresponding to the following tear proteins: defensins HNP-2 and HNP-3, lacritin and DMBT1.

## Discussion

### Major findings

A global gene expression analysis of pterygium showed distinct differences between primary pterygium and uninvolved conjunctiva. Several pathways were significantly affected in pterygium. These were: increase in the production of extracellular matrix, structural proteins, mitotic proteins, and protein involved in tissue invasion.

Recurrent pterygia demonstrated a different signature composed of other perturbed genes. For example, the COL4A6 (AL031177) and the RAB6B (BC002510) were down- and up-regulated respectively in recurrent pterygia compared to primary pterygia. The former encodes for one of the 6 subunits of collagen IV, a major component of the basement membrane, whereas the latter is a RAS family oncogene. The finding suggests that recurrence is a distinct biological phenomenon from the formation of primary pterygium, even though in general, we did not detect obvious microscopic changes between primary and recurrent pterygia.

Microarray analysis of gene expression is a useful approach for understanding the molecular mechanism of disease. We used both qPCR and immunohistochemistry to show that both RNA level and protein levels of specific mediators were dysregulated, validating the results from the gene microarray approach. Some 22 genes corresponding to tear proteins detected in pterygium subjects were significantly up or down-regulated in pterygium relative to conjunctival tissue. Many of the processes discovered in this study are highly novel and were not previously associated with pterygium, for example, immunohistochemistry show that cell adhesion molecules (ie., CD24) may be increased but abnormally localized in the nuclei in pterygium epithelium and therefore cell adhesion properties may be disturbed. Another example is the evidence for Goblet cell dysfunction. In pterygium, there was elevation of transcript and protein expression of MUC5AC and MSMB. MUC5AC is a well-known marker of conjunctival mucous-secreting Goblet cells,[25] and MSMB is a cutaneous squamous cell carcinoma related protein[26] that is associated with respiratory tract Goblet cells.[27]

### Comparison with previous studies

The data from a previous study utilized only 2 primary and 1 recurrent pterygia, with no stratification of data between the clinical sub-types.[16] This study[16] highlighted the up-regulation of 29 genes common to primary and recurrent pterygium. Our results supported the dysregulation of 19 of these genes in pterygium (Table 6), for example, TRAP100, MIP-4, RBP-1, MAP-17 and PECAM1. One apparent discrepancy was that the CLIC2 was up-regulated in our study, but significantly down-regulated in *John-Aryankalayil et al*.[16] Discrepancies reflect differences in study methodology, for example, this study[16] used an older microarray chip HG_U95Av2, containing considerably less probes than the U133A; and the earlier study may have differences in probe, chip or gene-level normalizations which were not reported. Differences in normalization may account for the differences in the folds of change in specific genes between different studies. In *John-Aryankalayil et al's *study, although a few up-regulated genes from different functional categories were tabulated, there was no attempt to evaluate the patterns of expression or relative contribution of broad biological processes.[16] Unlike this study, we did not restrict to listing differentially expressed genes by fold change.

**Table 6:** Comparison of microarray analysis between two studies

**Gene**	**Fold change in *John-Aryankalayil et al***	**Fold change in our study**
**Significantly regulated in current study (p < 0.05)**		

SILV (silver homolog)	2.2	2.3
CD31 (PECAM1)	2.1	2.2
C5orf13	2.5	2.2
RBP1	3.6	2.0
Von Willebrand factor	2.5	2.0
RAB31	2.0	1.7
Clathrin (CLTB)	2.4	1.7
DAB2	2.1	1.5
S100 A9 (calgranulin B)	2.2	1.4
MAP 17	2.4	1.4
MIP-4	4.9	1.4
ECM1	3.2	1.3
COMP	2.0	1.3
versican	3.3	1.2
TRAP100	5.0	1.2
CD32 receptor	2.9	1.2
mRNA, clone AL050154	2.8	1.1
CLIC2	0.5	1.1
Prostaglandin F2alpha receptor	3.2	1.1

**Not significantly regulated in current study (p > 0.05)**		

NDRG4	3.1	1.1
Carbonic anhydrase CA1	0.5	1.1
ABCG1	2.3	1.1
ADPRTL2	2.3	1.1
Casein kinase II	2.5	1.0
Collagen III	4.1	1.0
Fibronectin	8.5	1.0
LZTR1	2.2	1.0
Collagen VI	2.1	1.0
Myosin heavy chain II	3.1	0.9
FUBP	0.5	0.9
Ephrin-A1	0.5	0.9
Per1	0.5	0.9
Lipocalin 2	2.4	0.9

Previous studies[28,29] have shown that gene expression profiles in tumor and wound response were similar, lending support to our opinion that wound response is the major theme in global pterygial gene expression.

### Possible mechanisms of pterygia formation

We believe that our data support a predominantly wound healing pattern of gene expression in pterygium. Genes encoding for extracellular matrix, structural and adhesion molecules including wound healing related proteins, collagen subtypes, keratin 6A and fibronectin were significantly up-regulated in pterygium. Other up-regulated proteins like small proline rich protein 1B (SPRR1B), CD24, S100 calcium binding protein, SPARC, TFF1, SPRR1B and SERPINB13 also govern the wound healing process and cornification of epithelium, again, supporting the hypothesis of aberrant wound response in pterygium. Fibronectin alternatively splice transcript with EDA, up-regulated in our pterygial specimens, was shown to be up-regulated in wound healing.[30] In order to further validate this finding, semi-quantitative PCR was performed on paired pterygial and conjunctival tissue specimens from 3 patients to examine the level of fibronectin transcript with EDA. The results show that pterygium expressed higher levels of fibronectin transcript with EDA compared to uninvolved conjunctiva (Figure 8C).

Our data also suggest that in pterygia formation, there may be increased cellular proliferation and motility on one hand, and reduced cell death on the other. Genes up-regulated in pterygium include fibronectin (FN1), CEACAM5 (CEA), CD24, SPARC, MSMB and TFF1. CEACAM5 (CEA), a common cancer marker, was up-regulated 3.8-fold in pterygium. SPARC was known to have anti-adhesive property, and induced cancer cell motility.[31] The transcripts coding for microseminoprotein (MSMB) or PSP94[32] was up-regulated 3.6-fold, whereas transcripts for calcium binding protein S100A8 was up-regulated 5.7-fold in pterygium. Interestingly, in our analysis of tears from patients with pterygium, S100[18] proteins were also detected. Trefoil factors promote restitution of epithelial cells and are abundantly secreted onto the mucosal surface rapidly after mucosal injury.[33] In contrast to the above, genes coding for apoptosis (TGM2, IGFBP3 and DUSP1) were down-regulated in pterygium, reinforcing the over-proliferative tendency in pterygium.

The stress-inducible transcription regulator genes including ATF3, BTG2, EGR1, ERG2, FOS, JUN, NR4A1 and NR4A2 were surprisingly, down-regulated in pterygium relative to uninvolved conjunctiva. One has to bear in mind however, that our tissue specimens may represent chronic UV stimulation. The expression of these transcription factors may very well be elevated shortly after the commencement of the initial stimulus.

The transcript level of immunoglobulin subunit in pterygium was relatively lower as compared to uninvolved conjunctiva. This does not support a role for B cell mediated immunity in pterygium formation. However, the co-expression of some immunoglobulin genes (Figure 4) may have some biological significance, such as an immunological basis for the inappropriate wounding response.

### Possible mechanisms of pterygia recurrence

One hypothesis is that a low level of inflammation may stimulate epithelial and fibroblast cell migration due to factors such as SPN [see Additional file 3A]. This allows continuation of inflammatory factors and cytokines to be produced, potentiating a cycle [see Additional file 3B]. Factors such as SPN are unique to recurrence only, since they were up-regulated compared to primary pterygium cases.

In an alternative hypothesis, the formation of an exuberant scar or a 'go' signal, may or may not be coupled with a reduction of 'stop' or inhibitory signals. One such 'stop signal' may be the MSMB protein. Any up-regulation of this anti-metastatic gene in pterygium could give an inhibiting signal to slow the growth rate in pterygium. When pterygium is surgically removed, this inhibiting factor may also be reduced, encouraging the remnants of fibrous tissue to proliferate more aggressively, attaining the phenotype of recurrent pterygium. This may also explain the success of conjunctival autographs as adjunctive procedure after excision of the lesion in the prevention of recurrence. The graft tissue may 'replenish' any surgically-induced loss of 'stop' signals. In support of this hypothesis, the level of MSMB was significantly depressed in recurrent compared to primary pterygia.

### Strengths and Limitations

The strengths of this study include the use of a variety of analytical approaches to interpret the microarray data. The tissue specimens have been harvested in a center which manages a high volume of such disease. Samples have been processed in a very standardized fashion, obtained from patients with accurate clinical diagnoses.

One limitation of the study is that we did not evaluate p53 and related genes such as the Vasoendothelial growth factor (VEGF). Pterygia specimens contained a mixture of cell types whereas un-involved conjunctiva consisted largely of epithelial tissue. Therefore, any observations concerning differential gene expression could be related to differences in composition of cells as well as to pathological processes.

We did not notice regional differences in the intensity of CD24 immunofluorescent staining along the length of the pterygium epithelium on longitudinal section [see Additional file 7]. However, we cannot conclusively state that there is no difference in the pattern of CD24 expression between the head and the body of the pterygium. Since the limits of the pterygium body was defined clinically by the surgeon, we were uncertain if the bulk of the pterygium body was available for immunohistology.

Since we did not obtain temporally located pterygia in this study, our findings may not be applicable to this less frequently occurring type of pterygia.

Specimens analysed were at the later stage of pterygium formation when surgical removal was needed. No data at the early stage of disease were available for investigation.

In addition to these limitations, there are inherent limitations with hieuristic and even more deterministic methods of analyzing global gene expression. For example, the results of the k-means clustering depend arbitrarily on the initial number of clusters and the number of iterations. However, we attempt to reduce the effects of these shortcomings by employing a variety of different data-mining techniques and interpreting results as a whole and not in isolation. We drew conclusions based on findings that are consistent between different procedures and refrained from over-interpretation of isolated anomalies.

If changes in global gene expression in cases of recurrent pterygium do not occur until a very late time point, it may not be possible to predict recurrence of the condition by analysing the tissue obtained during the time of first excision. However, the gene expression data may still be useful for understanding the biology of recurrence. The tear protein analysis[18] also has limitations. It is difficult to determine whether the detected proteins were physiological, related to the surgical trauma, or arising from the disease. Nevertheless the 22 significantly up-regulated genes that have products in human tears may be potential biomarkers for the disease.

### Potential applications

This study illustrates that an unbiased global gene expression approach is useful to address the disease mechanisms in a controversial condition. Further studies on more specimens may allow the use of a sub-set of genes to prognosticate lesions. Non-surgical treatment for pterygium currently does not exist. Since wound healing and matrix dysregulation is a major theme, known modulators of wound healing may be explored in pterygium in a targeted way, using topical application. Other novel processes require further evaluation. Studies in the regulation of cell adhesion by CD24 and mucous processing pathways are required to understand pterygium formation. Studies are also required to elicit the origin of the overlying epithelium in pterygium tissue.

Nevertheless, our study suggests that after resection of the initial tumor, clinicians can modify treatment in selected patients based on objective criteria after analysing tumor gene expression or tumor proteins.

## Conclusion

Based on differential gene profiling in pterygium, it can be concluded that an aberrant wound healing process is the major pathogenetic process in pterygial formation. The other secondary processes such as cornification and attempted barrier formation may be compensatory in nature. This contrasts sharply with frank malignant neoplasms, where gene expression signatures are dominanted by increased proliferation, reduced apoptosis, cell cycling anomalies and genomic instability. The existence of an aggressive phenotype to account for post excision recurrence may be related to an imbalance of growth signals rather than due to mere prolongation of the stimulus that initiated primary pterygial formation.

## Abbreviations

CT: threshold cycle; (c)DNA: (complementary) deoxyribonucleic acid; ECM: extracellular matrix; FDR: false discovery rate; GEO: Gene expression omnibus; GSEA: Gene set enrichment analysis; OCT: optimal cutting temperature; (q) PCR: (quantitative) polymerase chain reaction; RMA: Robust Multi-array Average; (c)RNA: (complementary) ribonucleic acid.

## Competing interests

The authors declare that they have no competing interests.

## Authors' contributions

LT drafted and revised the manuscript, performed the analysis and coordinated study. JC carried out the molecular studies and microarray experiments, HY carried out the analysis on GSEA and provided help with bioinformatics, LPKA conceived of the study, coordinated study, revised the manuscript. DTHT critically reviewed manuscript, provided patient materials and RB initiated the study and formulated the design, revised the manuscript and approved the submission.

## Pre-publication history

The pre-publication history for this paper can be accessed here:



## Supplementary Material

Additional File 1 **Clinical appearance of pterygium on color photographs.** A. Primary pterygium and B. Recurrent pterygium. Note the excessive scarring that is typical of recurrent pterygium. Click here for file

Additional File 2 **Primary and recurrent pterygium specific genes.** Heat map showing pterygium specific and recurrence specific genes. Note that in recurrent pterygium a larger number of genes were relatively down-regulated (green) compared to primary pterygium and un-involved conjunctiva, and a smaller but still large number of genes were up-regulated (red) compared to primary pterygium and un-involved conjunctiva. Click here for file

Additional File 3 **Postulated pterygium recurrence pathways.** These pathways A and B were obtained from pathway analysis. Red symbols: genes up-regulated in recurrent pterygia compared to primary pterygia. Click here for file

Additional File 4 **Immunofluorescence image showing staining for CD24 in the pterygium epithelium.** Click here for file

Additional File 5 **Relationships in the pathway studio 5.0 analysis for up-regulated genes.** Click here for file

Additional File 6 **Relationships in the pathway studio 5.0 analysis for down-regulated genes.** Click here for file

Additional File 7 **Primers used for PCR.** Click here for file
