# Supplementary material for: Distinct gene subsets in pterygia formation and recurrence: dissecting complex biological phenomenon using genome wide expression data
Source: BMC Med Genomics. 2009 Mar 10;2:14. doi: 10.1186/1755-8794-2-14 (PMC2670830; doi:10.1186/1755-8794-2-14)
Supplement: Additional File 1 — Clinical appearance of pterygium on color photographs. A. Primary pterygium and B. Recurrent pterygium. Note the excessive scarring that is typical of recurrent pterygium. [file 1755-8794-2-14-S1.doc]

## Additional File 1. Primers used for PCR

| **Gene** | **Primers** | **Amplicon size** |
| --- | --- | --- |
| ATF3 | CCATCACAAAAGCCGAGGTA  GCCTTCAGTTCAGCATTCACA | 166 |
| BTG2 | TGTTGCGTGCTTGAGTCTGTG  TTGCTAACCTTGCTTGCTCCC | 139 |
| Jun | TGCCAACTCATGCTAACGCA  TCTCCGTCGCAACTTGTCAA | 126 |
| Egr1 | CACCTGACCGCAGAGTCTTTT  CCACAAGGTGTTGCCACTGTT | 156 |
| IGFPB3 | AAAAAGCAGTGTCGCCCTTCC  TCCACATTAACCTTGCGGCAG | 160 |
| GADD 45B | AATTGCAACATGACGCTGGA  TCAATGGCCAAGAGGCAGA | 194 |
| LAMB1 | AAAGCCATGGACTTGGACCA  TTTGCCTCATCTGCCCTCA | 101 |
| EFEMP1 | TAACTTTGTCATCCGGCGGA  CATGCAAAGGATCCCCGTAA | 192 |
| COL6A | CCCTCATCCAAAGCATCAAAG  GCAGTTGCTCTCAGCAATGGT | 185 |
| SERPINB13 | GCAGCCGATGAAAGTCGAAA  TAAACTCCCTGTCCCATTGCC | 157 |
| MAL | ACATAATTGGAGCCCACGGTG  CGGCAGCAATGTTTTCATGG | 168 |
| AQP1 | TCTTCCGTGCCCTCATGTACA  TTCACACCATCAGCCAGGTCA | 130 |
| ASPN | GGTGACGGTGTTCCATATCAGA  TTCCTAGGCCCAGCCTTTGTA | 164 |
| FN1 | TTCATGTCATCCTGTTGGCAC  TTCCCGAACCTTATGCCTCTG | 154 |
| COL1A1 | TTGCTTGAAGACCCATGCG  GCATTGCCTTTGATTGCTGG | 182 |
| SPRR1B | TGTTGCAGCATGAGTTCCCA  ATGCATGGTTCCTGAGGTGGA | 110 |
| KRT6A | TTTGCCTCCTTCATCGACAAG  ATGTACTGCTCGAACAACGGC | 134 |
| MSMB (PSP) | GGAGTCCTGCTTATCACAATG CTGATAGGCATGGCTACACAAT | 452,350 |
| Fibronectin EDA | AAACAGAAATGACTATTGAAGGCT TG  AGAGCATAGACACTCACTTCATAT TT | 604,334 |
| Fibronectin EDB | ATTACTGGTTATAGAATTACCACAACC  TAATATCAGAAAAGTCAATGCCAGTTG | 775,502 |

##### 
