# Supplementary material for: Distinct gene subsets in pterygia formation and recurrence: dissecting complex biological phenomenon using genome wide expression data
Source: BMC Med Genomics. 2009 Mar 10;2:14. doi: 10.1186/1755-8794-2-14 (PMC2670830; doi:10.1186/1755-8794-2-14)
Supplement: Additional File 2 — Primary and recurrent pterygium specific genes. Heat map showing pterygium specific and recurrence specific genes. Note that in recurrent pterygium a larger number of genes were relatively down-regulated (green) compared to primary pterygium and un-involved conjunctiva, and a smaller but still large number of genes were up-regulated (red) compared to primary pterygium and un-involved conjunctiva. [file 1755-8794-2-14-S2.doc]

**Additional file 2. Relationships in the pathway studio 5.0 analysis for up-regulated genes.**

| **Type of relation** | **Relation** | **Description of relationship** | MedLine Reference |
| --- | --- | --- | --- |
| Expression | CDKN1A <+-- TP53 | Cell cycle arrest is mediated in part by the p53-mediated transcriptional activation of the cyclin-dependent kinase inhibitor p21 (WAF-1). | 11306502:1045 |
| Expression | ITGAM <+-- S100A8 | Coimmunoprecipitation studies showed that NIF associated with CD11b in NIF(+/+) PMNs. | 15831844:3 |
| Expression | FN1 ---> PECAM1 | Soluble AGEs in combination with advanced glycated fibronectin significantly enhanced the endothelial cell surface expression of ICAM-1, VCAM-1 and PECAM-1, whereas this was not the case for E-selectin. | 9844140:6 |
| Expression | ABCA1 <+-- PPARG | In contrast, PPARgamma depletion reduced the expression of ABCA1 and LXRalpha mRNAs. | 11785957:3 |
| Expression | TP53 --+> THBS1 | Additional studies indicate that the tumor suppressor p53 positively regulates the TSP-1 gene (31) . | 14559817:1037 |
| Expression | SMAD4 ---> THBS1 | Rather, Smad4 restoration influenced angiogenesis, decreasing expression of vascular endothelial growth factor and increasing expression of thrombospondin-1. | 10944227:4 |
| Expression | CDKN1A |--- JUN | Previously, we found that c-jun represses the tumor suppressor p21((Waf1/Cip1/Sdi1)) (p21) gene expression. | 10733944:0 |
| Expression | FN1 <--- CREB1 | Thus p38(MAPK)-dependent CREB activation may mediate ANG II- and LO product-induced FN expression and cellular growth in rat MC. | 12372774:7 |
| Expression | JUN --+> FN1 | AP-1 inhibition reduced fibronectin production in a dosage-dependent manner. | 12540631:1016 |
| Expression | FOS ---> GJA1 | The combinations of c-Fos and c-Jun proteins activated the Cx43 promoter while c-Jun alone had no effect on Cx43 promoter activity. | 12064606:5 |
| Expression | APOE <+-- NR1H3 | Macrophage lipid loading leads to ligand activation of LXRs and to induction of a pathway for cholesterol efflux involving the LXR target genes ABCA1 and apoE. | 11604492:1 |
| Expression | CEBPB ---> TNFRSF6 | Although analysis of the human Fas promoter region ( 8 ) reveals the existence of two putative NF-kappaB consensus sequences, NF-IL6 has been proposed as the transcription factor mediating Fas induction in the influenza virus infection model ( 61 ). | 10373514:1297 |
| Expression | CDKN1A <+-- PPARG | Urothelial cells treated with PPARgamma ligands showed drastic morphologic changes and cell cycle arrest at G0/G1 phase accompanied with increased mRNA level of a cyclin-dependent kinase inhibitor p21(WAF1/CIP1). | 12012326:8 |
| Expression | CEBPB ---> S100A8 | We also show that the MRP-8 promoter is activated by NF-IL6 and retinoids inhibit MRP-8 expression by antagonizing the enhancer action of NF-IL6. | 9325272:1031 |
| Expression | SMAD4 --+> MUC5AC | By co-transfection studies we show that Smad4 is essential for Muc5ac promoter activation and that it does not synergize with Smad2 or Smad3. | 14570593:9 |
| Expression | CDKN1A <+-- SMAD3 | The upregulation occurs through Smad2/3-dependent transcriptional activation of the p21/WAF1 promoter region. | 14762439:6 |
| Expression | PPARG ---> APOE | In view of the widespread use of these drugs in human patients, the important role for PPARgamma in modulating adipocyte function, the observation that apoE is made in adipocytes, and the important and diverse roles that have been ascribed to apoE in organismal homeostasis, we evaluated a role for PPARgamma agonists in regulating adipocyte apoE expression. | 15339912:1044 |
| Expression | CREB1 ---> MUC5AC | Next, to determine whether CREB plays a role in MUC5AC gene expression, we used forskolin (an activator of adenylate cyclase) and 3-isobutyl-1-methylxanthine (IBMX; an inhibitor of AMP phosphodiesterase). | 12690113:1169 |
| Expression | CDKN1A <--- EGR1 | Zappia p21Cip1 Gene Expression Is Modulated by Egr1: A NOVEL REGULATORY MECHANISM INVOLVED IN THE RESVERATROL ANTIPROLIFERATIVE EFFECT J. | 11481417:1511 |
| Expression | PPARG ---> COL1A1 | It remains to be studied whether PPAR-gamma ligands inhibit alpha1(I) collagen gene expression in a similar mechanism. | 11606585:1222 |
| Expression | FOS <+-- RARA | In addition, RAR-alpha overexpression selectively inhibits the serum-stimulated expression of the c-fos gene, but does not affect the expression of a number of other serum- and polyomavirus-inducible genes including c-jun, junB, c-myc and actin. | 1380150:11 |
| Expression | APOE <--- ESR1 | Therefore, in the cortex, the mechanism for specific up-regulation of apoE was not ER mediated. | 11050140:1099 |
| Expression | ITGAM <+-- SP3 | Moreover, Sp3 activates the leukocyte integrin genes CD11c and CD11b in myelomonocytic cells ( 73 ). | 10722663:1325 |
| Expression | MITF ---> GP75 | Nevertheless, until now it has not been possible to show that MITF regulates the expression of the endogenous tyrosinase or Tyrp1. | 12859621:1 |
| Expression | ABCA1 <+-- RARA | Transactivation of the ABCA1 promoter by RAR was not dependent on cotransfection with RXR (Fig. 4B ), indicating that RAR activates the ABCA1 promoter, either by acting as a homodimer or as a heterodimer with endogenous RXR. | 14560020:1175 |
| PromoterBinding | ABCA1 ---- NR1H3 | Furthermore, ligands of the retinoid X receptor and LXR activate ABCA1 in ECs at levels of both promoter activation and mRNA induction. | 11788472:7 |
| PromoterBinding | CDKN1A ---- TP53 | During apoptotic cell death due to irradiation, p21cip-1/WAF-1 is up-regulated by a p53-dependent pathway that responds to DNA damage. | 10754295:3 |
| PromoterBinding | CDKN1A ---- SP1 | A , Sp1 overexpression leads to p21WAF1/Cip1 promoter-dependent repression in SMCs. | 12796485:1127 |
| PromoterBinding | CDKN1A ---- SMAD4 | Smad4 mediates TGF-beta1-induced up-regulation of p21Cip1 and growth arrest in MCA3D cells. | 10962574:2 |
| PromoterBinding | S100A8 ---- RARA | We also show that MRP-8 expression is retinoid inhibitable in cultured keratinocytes induced to differentiate with 10% serum or IFN-gamma, and that MRP-8 is inhibited by RAR but not by retinoid X receptor-specific retinoids in a dose-dependent manner. | 8959347:5 |
| PromoterBinding | CDKN1A ---- SP3 | These findings provide physical and functional evidence which strongly indicated that both Sp1 and Sp3 are responsible for TSA-induced transactivation of the murine p21WAF1 promoter in NIH3T3 cells. | 10321829:8 |
| PromoterBinding | SP1 ---- COL1A1 | Therefore, expression of the collagen alpha 1(I) gene may depend on the relative activities of NF-I and Sp1. | 2072909:6 |
| PromoterBinding | USF1 ---- CSTA | For example, the enhanced level of USF-1 mRNA may account for the increased expression of STF-1, because USF-1 specifically binds to the STF-1 promoter and can thereby function as an upstream regulator of STF-1 gene expression in HC islets ( 24 ). | 11500321:1228 |
| PromoterBinding | RARA ---- ESR1 | Therefore, both AF1 and AF2 of ER are necessary for estrogen induction of transcription from the RAR promoter. | 10076999:1117 |
| PromoterBinding | CEBPB ---- CREB1 | These experiments conclusively demonstrated that CREB binds to both sites in the LAP/C/EBP beta promoter with an affinity similar to that with the CREB consensus sequence. | 9199295:6 |
| PromoterBinding | JUN ---- RARA | These data underscore a specific role for RARgamma in inhibiting c-Fos expression, whereas either RARalpha or RARgamma affected c-Jun. | 10748128:1239 |
| PromoterBinding | MITF ---- TBX2 | USF and Mitf Bind the Tbx2 Promoter-- The results obtained so far suggest that the Tbx2 promoter is cell type-specific and contains a functional E-box motif that contributes substantially to promoter function. | 10770922:1154 |
| PromoterBinding | ABCA1 ---- SP1 | In conclusion, a functional complex of cis-elements within the proximal human ABCA1 promoter associated with the transcription factors Sp1/3, upstream stimulatory factors 1 and 2, and hepatic nuclear factor 1alpha has been characterized, which allows a subtle tissue-specific regulation of ABCA1 gene expression. | 11839742:1021 |
| PromoterBinding | CDKN1A ---- CEBPB | C/EBPbeta binds to the promoter of p21WAF1/CIP1, which is a powerful cell cycle inhibitor, thereby inducing its expression ( 49 ). | 12734198:1333 |
| PromoterBinding | FGFR1 ---- SP1 | Site directed mutagenesis and transfection studies indicated that both Sp1 sites are functional and both are required for FGFR1 promoter activity. | 11404014:6 |
| PromoterBinding | THBS1 ---- USF1 | Identification of an 18-bp Sequence of the TSP1 Promoter That Specifically Binds Glucose-induced USF1 and USF2A? To identify the nuclear proteins that bind to oligo V (55-bp region of TSP1 promoter A?932 to A?878), we performed a computer analysis using the GCG program, which revealed that this 55 bp contains putative binding sequences for several transcription factors, including NF-1, c-Jun, USF, heat shock transcription factor, C/EBP, and E12. | 15184388:1167 |
| PromoterBinding | ITGAM ---- SP1 | Despite being universally expressed ( 63 , 64 , 65 ), Sp1 is essential for myeloid-specific promoter activity in the CD11b gene ( 66 ). | 8943304:1322 |
| PromoterBinding | USF1 ---- TBX2 | In summary, the Tbx2 promoter contains a functional E-box element that is recognized both in vitro and in vivo by Mitf and USF1 . | 10770922:1214 |
| PromoterBinding | SP3 ---- COL1A1 | These results suggest that both Sp1 and Sp3 bind to the proximal COL1A1 promoter and stimulate its activity; however, their interaction with each other may result in repression of Sp1-induced COL1A1 transcription. | 9666093:8 |
| PromoterBinding | TNFRSF6 ---- USF1 | Two transcription factors, Upstream Stimulatory Factor (USF) and Sterol Regulatory Element Binding Protein-lc (SREBP-lc), seem to play a dominant and possibly cooperative role in regulating FAS transcription. | 15814457:5 |
| PromoterBinding | CEBPB ---- PPARG | ATF-2 regulated gene transcription of PPARgamma, which was synergistically enhanced by p38beta kinase and C/EBPbeta proteins expression. | 11243868:5 |
| PromoterBinding | JUN ---- THBS1 | We now demonstrate that the c-Jun-induced repression of TSP1 does not occur directly and does not require binding of c-Jun to the TSP1 promoter. | 10340386:2 |
| PromoterBinding | CEBPB ---- COL1A1 | Also, CCAAT/enhancer binding protein beta (C/EBPbeta) binds and activates the mouse alpha1(I) collagen promoter. | 10871045:1 |
| PromoterBinding | ABCA1 ---- USF1 | USF1, USF2, and Fra2 bind to the E-box motif and facilitate repression of the human ABCA1 promoter. | 11861672:1234 |
| PromoterBinding | SMAD3 ---- TAGLN | We found that TGF-beta1 transiently induces SRF and SM22 transcription, and that this process is accompanied by transient increases of SRF and Smad3 binding to the SM22 promoter. | 14654367:2 |
| PromoterBinding | SP1 ---- MUC5AC | Finally, we demonstrate that transcription factor Sp1 not only binds and activates MUC2 and MUC5AC promoters but also participates to their EGF- and TGF-alpha-mediated up-regulation. | 12077147:6 |
| PromoterBinding | EGR1 ---- FN1 | The activated Egr-1 may coordinate the expression of TGF-beta(1) and FN to regulate the development of silicosis. | 15033019:7 |
| PromoterBinding | GJA1 ---- TBX2 | We have shown that TBX2 binding suppresses Cx43 expression. | 14595187:3 |
| PromoterBinding | SP3 ---- MUC5AC | Electrophoretic mobility shift assays studies were thus carried out to identify new Sp1/Sp3 binding sites within MUC5AC promoter. | 12077147:1172 |
| PromoterBinding | CDKN1A ---- MITF | The results indicate that Mitf-mediated activation of p21(Cip1) expression and consequent hypophosphorylation of Rb1 will contribute to cell cycle exit and activation of the differentiation programme. | 15716956:4 |
| PromoterBinding | COL1A1 <+-- SP1 | Therefore, expression of the collagen alpha 1(I) gene may depend on the relative activities of NF-I and Sp1. | 2072909:6 |
| PromoterBinding | ABCA1 <--- SP1 | In conclusion, a functional complex of cis-elements within the proximal human ABCA1 promoter associated with the transcription factors Sp1/3, upstream stimulatory factors 1 and 2, and hepatic nuclear factor 1alpha has been characterized, which allows a subtle tissue-specific regulation of ABCA1 gene expression. | 11839742:1021 |
| PromoterBinding | FGFR1 <+-- SP1 | Site directed mutagenesis and transfection studies indicated that both Sp1 sites are functional and both are required for FGFR1 promoter activity. | 11404014:6 |
| PromoterBinding | ITGAM <--- SP1 | Despite being universally expressed ( 63 , 64 , 65 ), Sp1 is essential for myeloid-specific promoter activity in the CD11b gene ( 66 ). | 8943304:1323 |
| PromoterBinding | MUC5AC <+-- SP1 | Finally, we demonstrate that transcription factor Sp1 not only binds and activates MUC2 and MUC5AC promoters but also participates to their EGF- and TGF-alpha-mediated up-regulation. | 12077147:6 |
| PromoterBinding | SP1 ---> CDKN1A | Sp1 interacts with CDKN1A promoter | 15780936 |
| PromoterBinding | THBS1 <--- JUN | We now demonstrate that the c-Jun-induced repression of TSP1 does not occur directly and does not require binding of c-Jun to the TSP1 promoter. | 10340386:2 |
| PromoterBinding | CEBPB <+-- CREB1 | These experiments conclusively demonstrated that CREB binds to both sites in the LAP/C/EBP beta promoter with an affinity similar to that with the CREB consensus sequence. | 9199295:6 |
| PromoterBinding | COL1A1 <+-- CEBPB | Also, CCAAT/enhancer binding protein beta (C/EBPbeta) binds and activates the mouse alpha1(I) collagen promoter. | 10871045:1 |
| PromoterBinding | CEBPB ---> CDKN1A | C/EBPbeta binds to the promoter of p21WAF1/CIP1, which is a powerful cell cycle inhibitor, thereby inducing its expression ( 49 ). | 12734198:1333 |
| PromoterBinding | PPARG <+-- CEBPB | C/EBPdelta may function by synergizing with C/EBPbeta to enhance the level of PPARgamma expression. | 8754811:13 |
| PromoterBinding | RARA <--- ESR1 | Estrogen receptor (ER)-positive human breast carcinoma (HBC) cell lines express significantly higher levels of retinoic acid receptor alpha (RAR alpha) (isoform 1) mRNA than ER-negative HBCs. | 7585542:0 |
| Expression | SP1 --+> GJA1 | We identified a promoter region where cooperation between Ap1 and Sp1 elements was essential for TSA-induced cx43 transcription. | 16652385:5 |
| Expression | SP1 ---> APOE | TR4 also forms a complex with Sp1 to synergistically induce apoE expression via a region containing the TR4RE-DR0-apoE and the Sp1 binding site (-169 to -140bp). | 15670754:3 |
| Expression | SP1 --+> THBS1 | Overlapping Egr-1 and Sp1 sites function in the regulation of transcription of the mouse thrombospondin 1 gene. | 9799798:1561 |
| Expression | SP1 --+> FN1 | Induction of Sp1 expression in NEC14 cells effectively stimulated the promoter activity of the transfected FN promoter-CAT constructs. | 9566920:9 |
| Expression | SP1 ---| TAGLN | Consistent with this hypothesis, results of studies in cultured SMCs showed that: (1) PDGF-BB increased expression of Sp1; (2) PDGF-BB and Sp1 profoundly suppressed SM22alpha promoter activity as well as smooth muscle myosin heavy chain promoter activity through mechanisms that were at least partially dependent on the G/C cis element; and (3) a short interfering RNA to Sp1 increased basal expression and attenuated PDGF-BB induced suppression of SM22alpha. | 15486317:7 |
| PromoterBinding | SP1 ---| CDKN1A | Sp1 interacts with CDKN1A promoter | 15780936 |
| PromoterBinding | SP1 ---> FGFR1 | Site directed mutagenesis and transfection studies indicated that both Sp1 sites are functional and both are required for FGFR1 promoter activity. | 11404014:6 |
| Expression | FN1 <--- TP53 | HBxAg also antagonized the repression of the FN promoter by the tumour suppressor, p53. | 15230856:6 |
| PromoterBinding | CDKN1A <+-- TP53 | p53 interacts with p21 promoter | 15989967 |
| Expression | SPARC |--- CDKN1A | However, experiments utilizing the DNA synthesis inhibitor hydroxyurea show that the downregulation of osteonectin expression by bFGF is independent of the mitogenic properties of the growth factor. | 9530105:1174 |
| Expression | FN1 --+> TNFRSF6 | To determine whether FN adhesion altered CD95 expression, we examined CD95 cell surface and total protein levels in U937 cells. | 11859150:1157 |
| Expression | SPARC |--- THBS1 | Although c-Jun regulation of the TS1 gene was found at the promoter level, preliminary results strongly suggest that repression of SPARC and TS1 gene expression are mediated by a secreted factor. | 7988564:7 |
| Expression | SPARC ---> FGFR1 | We also report that in the presence of FGF-2, a factor which otherwise stimulates myoblast proliferation and the repression of terminal differentiation, both native SPARC and the Ca(2+)-binding SPARC peptide significantly promote (>60%) the differentiation of the MM14 murine myoblast cell line that expresses FGFR1 almost exclusively. | 14505356:2 |
