# Supplementary material for: Distinct gene subsets in pterygia formation and recurrence: dissecting complex biological phenomenon using genome wide expression data
Source: BMC Med Genomics. 2009 Mar 10;2:14. doi: 10.1186/1755-8794-2-14 (PMC2670830; doi:10.1186/1755-8794-2-14)
Supplement: Additional File 3 — Postulated pterygium recurrence pathways. These pathways A and B were obtained from pathway analysis. Red symbols: genes up-regulated in recurrent pterygia compared to primary pterygia. [file 1755-8794-2-14-S3.doc]

**Additional file 3. Relationships in the pathway studio 5.0 analysis for down-regulated genes.**

| **Type of relation** | **Relation** | **Description of relationship** | **MedLine Reference** |
| --- | --- | --- | --- |
| Expression | TP53 --+> PMAIP1 | Moreover, S-nitroso-HCy did not increase ROS generation, p53-dependent Noxa expression, and apoptosis. | 15561702:8 |
| Expression | TP53 ---> MYC | In addition, p53 has been shown to be a transcriptional modulator of c-myc expression. | 1737333:3 |
| Expression | CRSP3 --+> TXNIP | Loss or decreased expression of CRSP3 would result in diminished expression of TXNIP and KISS1, which would, in turn, allow cells to metastasize. | 12543799:1332 |
| Expression | TP53 --+> IGFBP3 | We found that the IGFBP3 promoter was activated by p53(DeltaNDeltaBD), which mimics a naturally occurring N- and C-terminally truncated human p53 isoform, and by p53AS, a C-terminally truncated murine p53 isoform generated through alternative splicing, but not by full-length human or murine p53. | 15713654:7 |
| Expression | TP53 --+> BTG2 | Recently, we have shown that BTG2/TIS21 gene expression was induced in response to DNA damage through a p53-dependent pathway. | 10657898:2 |
| Expression | CRSP3 --+> Kis1 | Quantitative real-time reverse-transcription PCR of clinical melanoma samples showed that loss of CRSP3 expression correlated with decreased KISS1 expression and increased metastasis. | 12543799:1024 |
| Expression | RARA ---> CYP26A1 | In Caco-2 cells not only all- trans -RA but also synthetic agonists of the RAR induced intestinal CYP26 gene expression and all- trans -RA metabolism as well. | 11302942:1007 |
| Expression | APOA1 <--- SP1 | Insulin induces transcription of the hepatic apolipoprotein AI (apo AI) gene by increasing Sp1 binding to the promoter. | 15375792:0 |
| Expression | NR5A2 <--- NR0B1 | The expression of Dax-1 was significantly impaired in knock-out mice of the Ftz-f1 gene, which encodes Ad4BP/ SF-1. | 10446902:7 |
| Expression | SP1 ---> CYP17 | Thus, GATA-4 or GATA-6 and Sp1 together regulate expression of P450c17 in adrenal NCI-H295A cells and methylation of P450c17, GATA-4 and GATA-6 silence the expression of P450c17 in placental JEG-3 cells. | 14988427:13 |
| Expression | JUN ---> COL1A2 | Overexpression of c-Jun enhanced in a dose-dependent manner the ability of halofuginone to inhibit the activity of a luciferase reporter construct under control of the -3200-bp to +54-bp COL1A2 promoter, whereas the expression of a dominant-negative c-Jun construct abolished this effect. | 12384935:9 |
| Expression | RARA --+> POU5F1 | Conversely, activation of Oct-3/4 promoter by RAR:RXR heterodimers was completely abolished by EAR-3/COUP-TFI and by ARP-1/COUP-TFII. | 7823919:9 |
| Expression | RARA ---> DUSP1 | Contrastive Roles of Three RAR Subtypes-- Since RAR is required for the retinoid-induced MKP-1 expression, we further examined roles of RAR subtypes in the regulation of MKP-1 . | 12186877:1185 |
| Expression | PSEN1 <--- NFIC | PS1/CTF Mediates PS1 Binding to Juxtamembrane Cytoplasmic E-cadherin. | 11226248:1095 |
| Expression | TP53 --+> DUSP1 | Activation of p53 induces the expression of MKP1 transcript and protein in several well defined p53-regulated cell systems. | 12890671:1043 |
| Expression | JUN ---| CYP7A1 | Overexpression of dominant-negative JNK1 or a transactivating domain mutant of c-Jun significantly blocked the ability of TCA to down-regulate CYP7A1 mRNA. | 11278771:1020 |
| Expression | EGR1 --+> NR4A1 | Furthermore, we show that Zif268 can activate transcription of the nur77 promoter, suggesting that it may play a role in the delayed-early expression of nur77. | 8413214:8 |
| Expression | JUNB --+> COL1A2 | Co-transfection of a junB stimulated the basal activity of the alpha2(I) collagen promoter 93-fold, respectively. | 7876214:1008 |
| Expression | NR5A2 --+> AFP | The alpha1-fetoprotein (AFP) gene is located between the albumin and alpha-albumin genes and is activated by transcription factor FTF (fetoprotein transcription factor), presumed to transduce early developmental signals to the albumin gene cluster. | 8668203:0 |
| Expression | NFIC --+> SFTPC | We find that NFI binding is required for transcription of the mouse SP-C promoter in lung cells and that the NFI-A1.1 isoform activates transcription of this promoter in HeLa cells. | 9407049:1041 |
| Expression | JUN <--- EGR1 | Egr-1 biosynthesis was activated by arsenite concentrations insufficient for the induction of c-Jun biosynthesis. | 15292961:6 |
| Expression | SCARB1 <--- NR5A2 | The orphan nuclear receptor liver receptor homolog 1 (LRH-1) and SR-BI are co-expressed in liver and ovary, suggesting that LRH-1 might control the expression of SR-BI in these tissues. | 12446566:1 |
| Expression | NR4A1 |--- NR0B1 | We demonstrate that DAX-1 represses the Nur77 transactivation by transient transfection assays. | 15155786:2 |
| PromoterBinding | MYC ---- SRF | The ternary complex factor-serum response factor complex then binds to the serum response element within the promoter of immediate early proto-oncogenes such as c-fos, c-jun, and c-myc. | 11882598:1108 |
| PromoterBinding | SCARB1 ---- SP1 | We demonstrate that Sp1 binds to three sites within the region of the rat SR-BI gene promoter. | 10801817:1036 |
| PromoterBinding | EGR1 ---- SP1 | Changes in the level of Sp1 phosphorylation are important for displacement by Egr-1 in the common binding site of the MT1-MMP promoter. | 12093818:1245 |
| PromoterBinding | SP1 ---- CETP | Mutations at this site lead to marked reduction in the in vitro transcriptional activity of the CETP promoter, thereby indicating that Sp1 is a key factor in the activation of CETP gene expression. | 12730302:1028 |
| PromoterBinding | EGR1 ---- SRF | Our data provide the first evidence that SRF is expressed in granulosa cells and binds the Egr-1 promoter. | 12554779:1265 |
| PromoterBinding | TP53 ---- JUN | Shreiber et al. (21) have recently shown that c-Jun directly down-regulates p53 expression through binding to a variant AP-1 site in the endogenous cellular p53 promoter. | 10470854:1162 |
| PromoterBinding | TP53 ---- SP1 | We demonstrate that the interaction of Vpr and Sp1 can potentiate p53 to enhance transcription of p21 via the GC-rich motif. | 15302882:1043 |
| PromoterBinding | TP53 ---- MYC | Many cellular oncoproteins, including c-myc, E1A, Ras, and E2F1, induce the stabilization and accumulation of p53. | 11457731:1158 |
| PromoterBinding | SP1 ---- IGFBP3 | Taken together, these results show that Sp1 phosphorylation and the modulation of the Sp1/Sp3/HDAC1 multiprotein complex play a pivotal role in the transcriptional activation of the IGFBP-3 promoter through the Sp1/GC-rich site by TSA. | 12200149:6 |
| PromoterBinding | TP53 ---- AFP | HNF-3 protein activates while p53 represses AFP transcription through sequence-specific binding within the previously identified AFP developmental repressor domain. | 9891062:2 |
| PromoterBinding | NR5A2 ---- CYP7A1 | FTF+/- mice express higher levels of CYP7A1 mRNA than wild-type mice. | 15014077:1273 |
| PromoterBinding | CETP ---- NR5A2 | The orphan nuclear receptor LRH-1 potentiates the sterol-mediated induction of the human CETP gene by LXR. | 12730302:1438 |
| PromoterBinding | SP1 ---- COL1A2 | Decoy Sp1 binding oligonucleotides inhibited COL1A2 promoter activity both in cultured fibroblasts and in vivo, in the skin of transgenic mice, which have integrated a mouse COL1A2 promoter/luciferase reporter gene construct. | 11348466:6 |
| PromoterBinding | NR5A2 ---- CYP19 | The overlapping but distinct expression patterns of LRH-1 and P450arom circumstantially support the recent finding that LRH-1 serves as a critical upstream regulator of P450arom gene expression in ovarian cells, but LRH-1 also may be a multifunctional steroidogenic factor in ovarian physiology. | 12672674:10 |
| PromoterBinding | SRF ---- ETR101 | The serum response element (SRE) is the major regulatory element located in the pip92 promoter which is recognized by the serum response factor (SRF) and ternary complex factor (TCF), a family of Ets-domain transcription factors [ 15 ]. | 10903500:1035 |
| PromoterBinding | JUN ---- RARA | These data underscore a specific role for RARgamma in inhibiting c-Fos expression, whereas either RARalpha or RARgamma affected c-Jun. | 10748128:1239 |
| PromoterBinding | JUN ---- SP1 | Chen et al. ( 54 ) identified a 20-kDa protein fraction referred to as Sp1-I that inhibited binding of Sp1 to the rat c- jun promoter. | 10722744:1366 |
| PromoterBinding | TP53 ---- PSEN1 | Presenilin 1 (PS1) expression is repressed by the p53 tumor suppressor. | 10805794:0 |
| PromoterBinding | MYC ---- SP1 | In addition, enforced expression of Sp3 repressed Sp1-mediated activation of c-myc. | 7753559:5 |
| PromoterBinding | TP53 ---- EGR1 | Moreover, chromatin immunoprecipitation analysis indicates that mutant p53 is physically associated with the EGR1 promoter. | 15548700:4 |
| PromoterBinding | NR5A2 ---- ABCC3 | The expression of FTF mRNA levels were also markedly enhanced in response to CDCA, and overexpression of FTF specifically activated the MRP3 promoter activity about 4-fold over the basal promoter activity. | 11590139:8 |
| PromoterBinding | RARA ---- PCK2 | In hepatocytes, gAF1/PCK1 is bound by a variety of nuclear receptors including COUP-TFs, HNF4, RAR/RXR, and PPARalpha that activate the PEPCK promoter ( 39-41 ). | 11399762:1060 |
| PromoterBinding | NR5A2 ---- CEL | Auwerx Liver Receptor Homolog 1 Controls the Expression of Carboxyl Ester Lipase | 12198243:1488 |
| PromoterBinding | JUNB ---- MYC | At this point, it is important to notice that although ChIP analysis was not able to detect binding of JunB to the c- myc promoter, EMSA experiments showed a small amount of this protein bound to the c- myc AP-1-containing EMSA probes. | 14523011:1155 |
| PromoterBinding | JUN ---- AFP | The Jun-induced repression does not require binding of c-Jun to the AFP promoter. | 7537266:1010 |
| PromoterBinding | SRF ---- SP1 | We tested if the Sp1 sites from the SRF promoter could bind both Sp1 and Egr-1 present in myotube nuclear extracts. | 9218459:1279 |
| PromoterBinding | MYC ---- EGR1 | Repression of c- myc and induction of IRF-1 , but not induction of egr-1 , is inhibited in M1 cells expressing PTP epsilonC. | 10859312:1140 |
| PromoterBinding | SP1 ---- HBB | Further analysis of this region demonstrates that multiple sequences are required for maximal enhancer activity; deletion of SP1, NF-E2, GATA-1 or USF binding sites significantly decrease beta-globin gene expression. | 8152905:4 |
| PromoterBinding | TITF1 ---- SP1 | Electrophoretic mobility shift assays revealed binding of Sp1 and Smad4 to the NKX2.1 promoter. | 15264213:5 |
| PromoterBinding | APOA1 ---- NR5A2 | Chromatin immunoprecipitation experiments revealed that LRH-1 binds to the human APO AI promoter in vivo. | 15218078:4 |
| PromoterBinding | SP1 ---- TFPI2 | Luciferase activity consistently increased after stimulation of JEG-3 cells by phorbol 12-myristate 13-acetate indicating that NF1, NF-kappa B and egr-1/Sp1 binding sites are crucial in inducible TFPI-2 expression. | 12757776:6 |
| PromoterBinding | EGR1 ---- TFPI2 | Luciferase activity consistently increased after stimulation of JEG-3 cells by phorbol 12-myristate 13-acetate indicating that NF1, NF-kappa B and egr-1/Sp1 binding sites are crucial in inducible TFPI-2 expression. | 12757776:6 |
| PromoterBinding | NR5A2 ---- POU5F1 | In vitro and in vivo results show that LRH-1 plays an essential role in the maintenance of Oct4 expression in ES cells at the epiblast stage of embryonic development, thereby maintaining pluripotence at this crucial developmental stage prior to segregation of the primordial germ cell lineage at gastrulation. | 15831456:6 |
| PromoterBinding | NR4A1 ---- NR4A2 | Nur77 and its close relatives Nurr1 and NOR-1 bind as monomers to a consensus binding site, the nerve growth factor induced protein I-B (NGFI-B)-binding response element (NBRE). | 12082103:1 |
| PromoterBinding | NR4A1 ---- CYP17 | A Nur77-binding site within the rat P450c17 gene promoter has been identified ( 62 ). | 15024051:1032 |
| PromoterBinding | SP1 ---- POU5F1 | The lactoferrin ( 37 ) and Oct-4 (38) gene promoters also contain multiple Sp1 binding sites in close proximity to nuclear receptor response elements. | 7608198:1195 |
| PromoterBinding | TP53 ---- PMAIP1 | Here we show that, like Bax, Brn-3a antagonizes p53-mediated transcription of another proapoptotic target, Noxa, significantly reducing transactivation of the Noxa promoter by p53. | 15598651:3 |
| PromoterBinding | POU5F1 <--- SP1 | The lactoferrin ( 37 ) and Oct-4 (38) gene promoters also contain multiple Sp1 binding sites in close proximity to nuclear receptor response elements. | 7608198:1195 |
| PromoterBinding | SRF <--- SP1 | We tested if the Sp1 sites from the SRF promoter could bind both Sp1 and Egr-1 present in myotube nuclear extracts. | 9218459:1279 |
| PromoterBinding | COL1A2 <+-- SP1 | In conclusion, three regions of Sp1 binding were identified and are required for optimal activity of the alpha 2(I) collagen promoter. | 9143363:8 |
| PromoterBinding | MYC <--- SP1 | In addition, enforced expression of Sp3 repressed Sp1-mediated activation of c-myc. | 7753559:5 |
| PromoterBinding | SP1 ---> HBB | Further analysis of this region demonstrates that multiple sequences are required for maximal enhancer activity; deletion of SP1, NF-E2, GATA-1 or USF binding sites significantly decrease beta-globin gene expression. | 8152905:4 |
| PromoterBinding | IGFBP3 <--- SP1 | Taken together, these results show that Sp1 phosphorylation and the modulation of the Sp1/Sp3/HDAC1 multiprotein complex play a pivotal role in the transcriptional activation of the IGFBP-3 promoter through the Sp1/GC-rich site by TSA. | 12200149:6 |
| PromoterBinding | JUN <--- SP1 | Chen et al. ( 54 ) identified a 20-kDa protein fraction referred to as Sp1-I that inhibited binding of Sp1 to the rat c- jun promoter. | 10722744:1366 |
| PromoterBinding | CETP <--- SP1 | This finding demonstrated that the -690 Sp1/Sp3 binding site repressed human CETP promoter activity. | 12730302:1119 |
| PromoterBinding | SCARB1 <--- SP1 | We demonstrate that Sp1 binds to three sites within the region of the rat SR-BI gene promoter. | 10801817:1036 |
| PromoterBinding | SP1 --+> TP53 | We demonstrate that the interaction of Vpr and Sp1 can potentiate p53 to enhance transcription of p21 via the GC-rich motif. | 15302882:1043 |
| PromoterBinding | SP1 ---> TFPI2 | Luciferase activity consistently increased after stimulation of JEG-3 cells by phorbol 12-myristate 13-acetate indicating that NF1, NF-kappa B and egr-1/Sp1 binding sites are crucial in inducible TFPI-2 expression. | 12757776:6 |
| PromoterBinding | TITF1 <--- SP1 | Electrophoretic mobility shift assays revealed binding of Sp1 and Smad4 to the NKX2.1 promoter. | 15264213:5 |
| PromoterBinding | CYP19 <--- JUN | JUN (cJUN) interacts with the CYP19A1 (aromatase PII) promoter | 15688015 |
| PromoterBinding | JUN ---> AFP | The Jun-induced repression does not require binding of c-Jun to the AFP promoter. | 7537266:1010 |
| PromoterBinding | JUN ---| TP53 | The AP-1 motif in the human p53 promoter binds c-Fos and c-Jun and the NF-kappaB motif binds p50(NF-kappaB) and p65 RelA. | 10348347:4 |
| PromoterBinding | PMAIP1 <--- MYC | c-Myc interacts with NOXA promoter | 15856024 |
| PromoterBinding | TP53 <+-- MYC | Furthermore, we found that the c-myc and bcl-2 genes cooperate to inhibit p53 functions. | 8016082:3 |
| PromoterBinding | TP53 <--- MYC | Myc interacts with the Trp53 (p53) promoter | 15735755 |
| PromoterBinding | NFIC ---| CYP17 | With respect to the CYP17 promoter, recent data in adrenal H295 cells have shown that NF-1C binds the CYP17 promoter ( 29 ); however, NF-1C-dependent regulation of the CYP17 gene has not been characterized. | 14684846:1050 |
| PromoterBinding | NFIC ---> TITF1 | Thus, NFI binding to these elements is critical for constitutive expression of TTF-1; TG decreases NFI binding to the NFI elements in association with TG repression. | 11046146:3 |
| PromoterBinding | NFIC ---> COL1A2 | In this study, we determined if acetaldehyde influences the binding of NF-I to the alpha 2(I) collagen promoter. | 7768510:3 |
| PromoterBinding | NFIC ---| Mt1 | We considered nuclear factor I (NFL) protein as a potential repressor, as three half-sites for NFI binding are present on MT-I promoter and NFI is known to downregulate several cellular gene promoters. | 11444530:1 |
| PromoterBinding | NFIC ---> PCK2 | Purified NFI binds the P1 site of the PEPCK gene promoter ( 6 ). | 9593667:1027 |
| PromoterBinding | NFIC ---> CEL | During development of the mouse mammary gland, binding of NF1-C2 to the CEL gene promoter is induced at midpregnancy, in correlation with the induction of CEL gene expression. | 11877413:1014 |
| PromoterBinding | NFIC ---| LOC287167 | Two established cell lines, which express alpha-globin differentially, were therefore compared for differences in binding of NFI at the alpha-globin promoter in vivo . | 7642653:1008 |
| PromoterBinding | EGR1 --+> TP53 | These findings showed direct interaction of EGR-1 with the p53 promoter element. | 9242687:1181 |
| PromoterBinding | SP1 <+-- EGR1 | Changes in the level of Sp1 phosphorylation are important for displacement by Egr-1 in the common binding site of the MT1-MMP promoter. | 12093818:1246 |
| PromoterBinding | MYC <--- EGR1 | In addition, EGR-1 may be involved in the regulation of the c-myc gene, which contains an EGR-1 site ( 17 ) and also exhibits delayed induction( 4 ) . | 8576248:1241 |
| PromoterBinding | TFPI2 <--- EGR1 | Luciferase activity consistently increased after stimulation of JEG-3 cells by phorbol 12-myristate 13-acetate indicating that NF1, NF-kappa B and egr-1/Sp1 binding sites are crucial in inducible TFPI-2 expression. | 12757776:6 |
| Expression | SP1 --+> LOC287167 | Data from Pondel et al. ( 61 ) indicate that Sp1 is critical for the chromatin-dependent expression of the human alpha-globin gene, and work from Marin et al. ( 62 ) shows that embryonic globin genes were expressed at reduced levels in Sp1 homozygous knockout mice. | 11304527:1324 |
| Expression | SP1 --+> TGM2 | Deletion analyses indicated that putative Sp1 binding motifs may be responsible in the TGM2 gene( 56 ) . | 8626812:1234 |
| Expression | SP1 --+> CYP26A1 | In summary, analysis of the mouse P450RAI promoter revealed the presence of a highly conserved RARE whose activity depends on an upstream Sp1/Sp3 element. | 10976925:1222 |
| Expression | SP1 ---> Mt1 | Sp1 and MLTF stimulate basal expression of the MT-I gene ( 21 , 29 ). | 11713267:1210 |
| Expression | SP1 --+> ABCC3 | These studies indicate that Sp1 and Sp3 may be involved in the regulation of the rat Mrp3 gene. | 11846400:4 |
| Expression | SP1 ---> Kis1 | Together, our results indicate that AP-2alpha and Sp1 are strong transcriptional regulators of KiSS-1 and that loss or decreased expression of AP-2alpha in breast cancer may account for the loss of tumor metastasis suppressor KiSS-1 expression and thus increased cancer metastasis. | 16260418:7 |
| Expression | MYC |--- TP53 | We have addressed the possibility that elevated mutant p53 expression is due to deregulated c-Myc expression. | 7969121:3 |
| Expression | TP53 ---> COL1A2 | The results of transient transfection assays showed that ectopic expression of p53 resulted in repression of basal COL1A2 promoter-driven transcriptional activity and prevented its stimulation induced by TGF-beta ( Fig. 1 B , left panel ). | 15345715:1121 |
| Expression | TP53 ---> SFTPC | To achieve this goal, a "dominant negative" mutant form of p53 was expressed from the human surfactant protein C (SPC) promoter. | 9610073:3 |
| Expression | Mt1 <+-- TP53 | In an attempt to analyse in vivo the effects of p53 activity, we have generated transgenic mice expressing the wild-type p53 under the control of the metallothionein I promoter. | 10597255:1 |
| PromoterBinding | TP53 ---> JUN | Fra-1 and c-jun were induced by p53, resulting in increased AP-1 levels. | 9661907:4 |
| PromoterBinding | TP53 ---| AFP | Loss of p53 gene was related to the serum alpha-fetoprotein (AFP) level and the tumor size (P < 0.05). | 12760798:6 |
| PromoterBinding | TP53 ---| PSEN1 | Presenilin 1 (PS1) expression is repressed by the p53 tumor suppressor. | 10805794:0 |
| PromoterBinding | TP53 ---| EGR1 | Moreover, chromatin immunoprecipitation analysis indicates that mutant p53 is physically associated with the EGR1 promoter. | 15548700:4 |
| PromoterBinding | TP53 --+> PMAIP1 | Trp53 (p53) interacts with the Pmaip1 (Noxa) promoter | 15750633 |
