# Supplementary figures and images for: Distinct gene subsets in pterygia formation and recurrence: dissecting complex biological phenomenon using genome wide expression data
Source: BMC Med Genomics. 2009 Mar 10;2:14. doi: 10.1186/1755-8794-2-14 (PMC2670830; doi:10.1186/1755-8794-2-14)

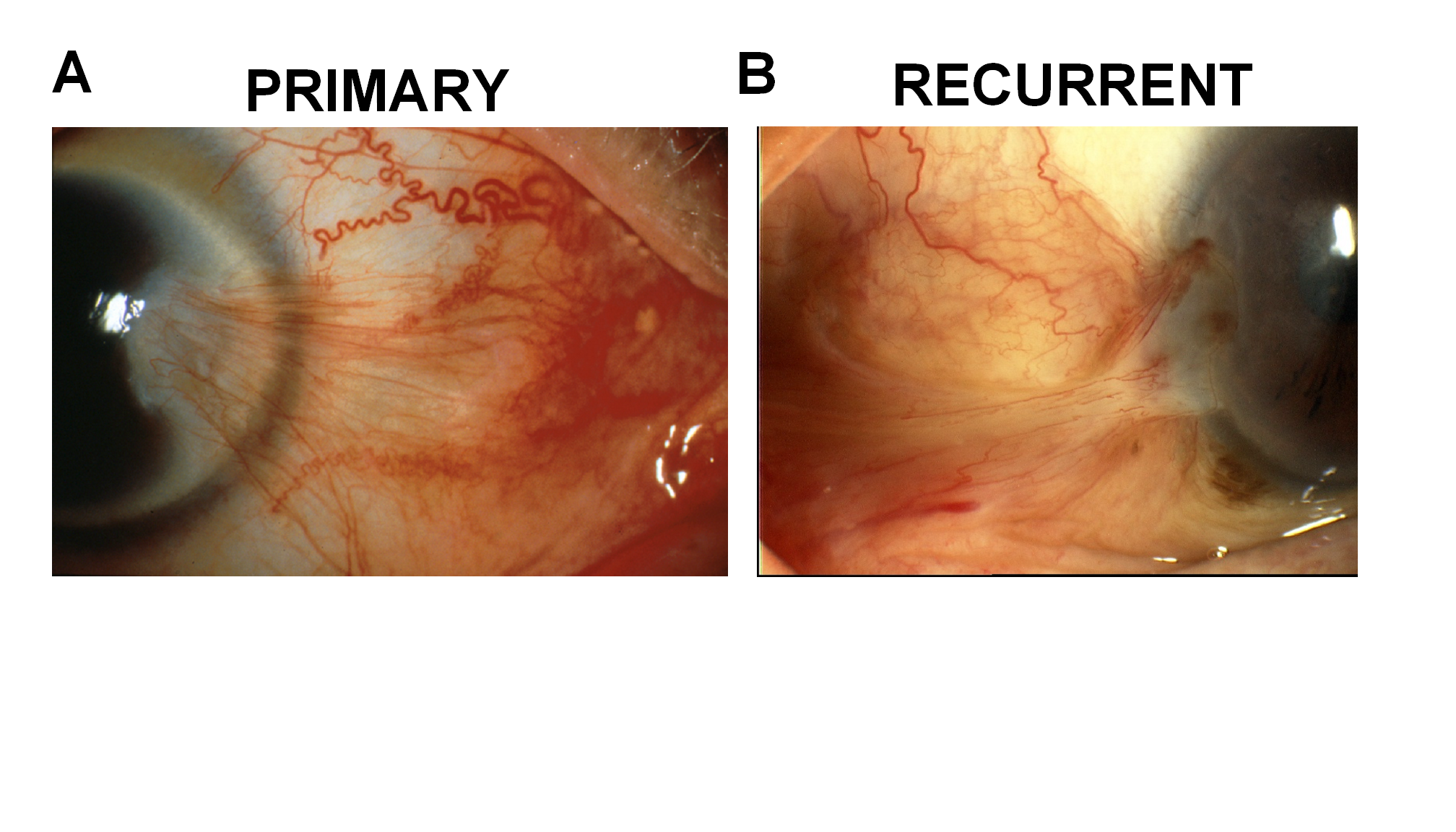

Supplement: Additional File 4 — Immunofluorescence image showing staining for CD24 in the pterygium epithelium. [file 1755-8794-2-14-S4.tiff]

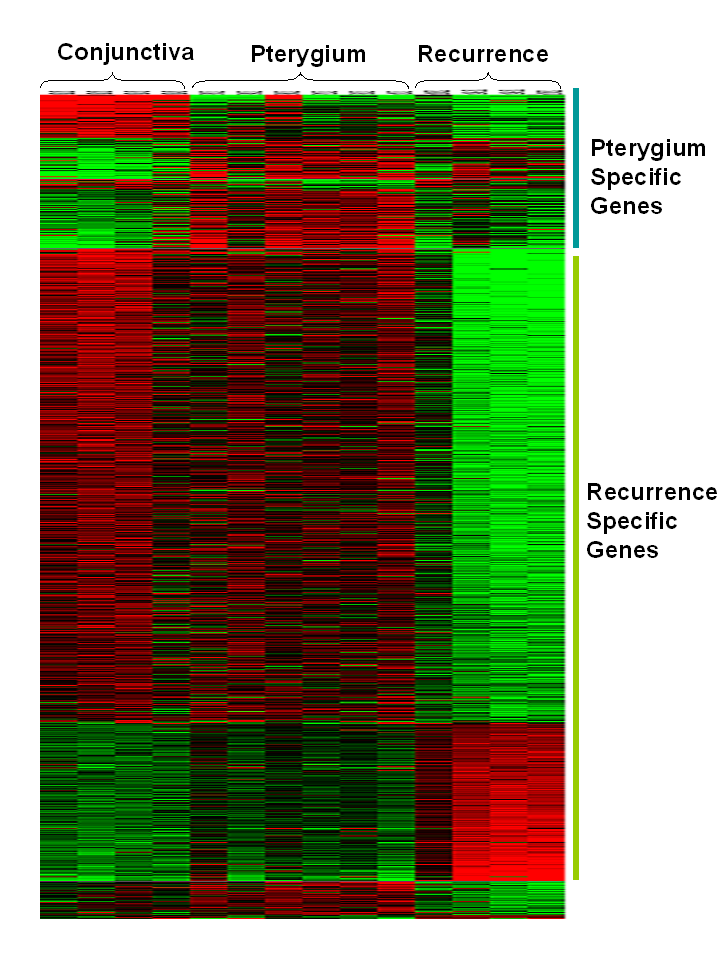

Supplement: Additional File 5 — Relationships in the pathway studio 5.0 analysis for up-regulated genes. [file 1755-8794-2-14-S5.tiff]

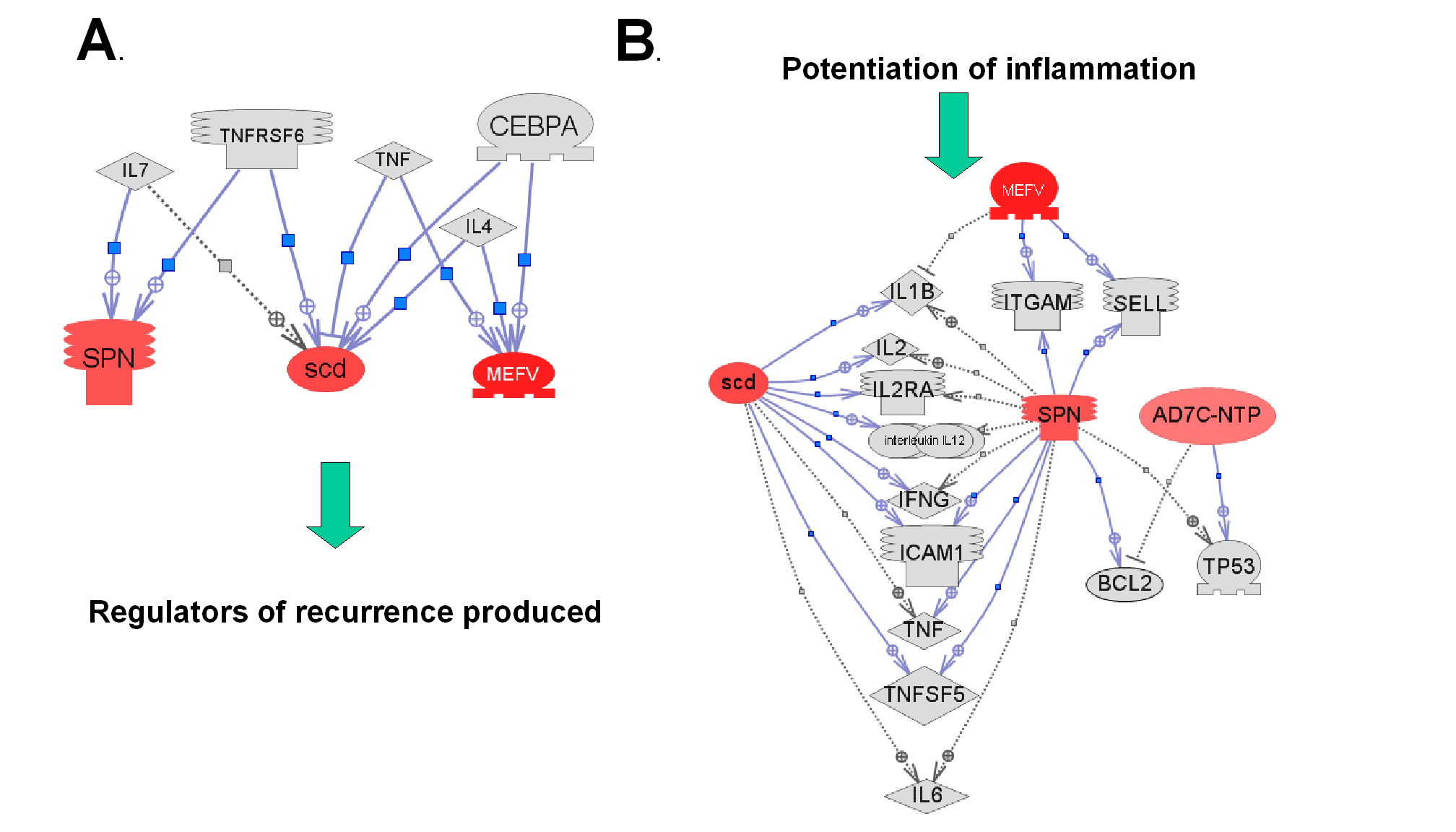

Supplement: Additional File 6 — Relationships in the pathway studio 5.0 analysis for down-regulated genes. [file 1755-8794-2-14-S6.tiff]

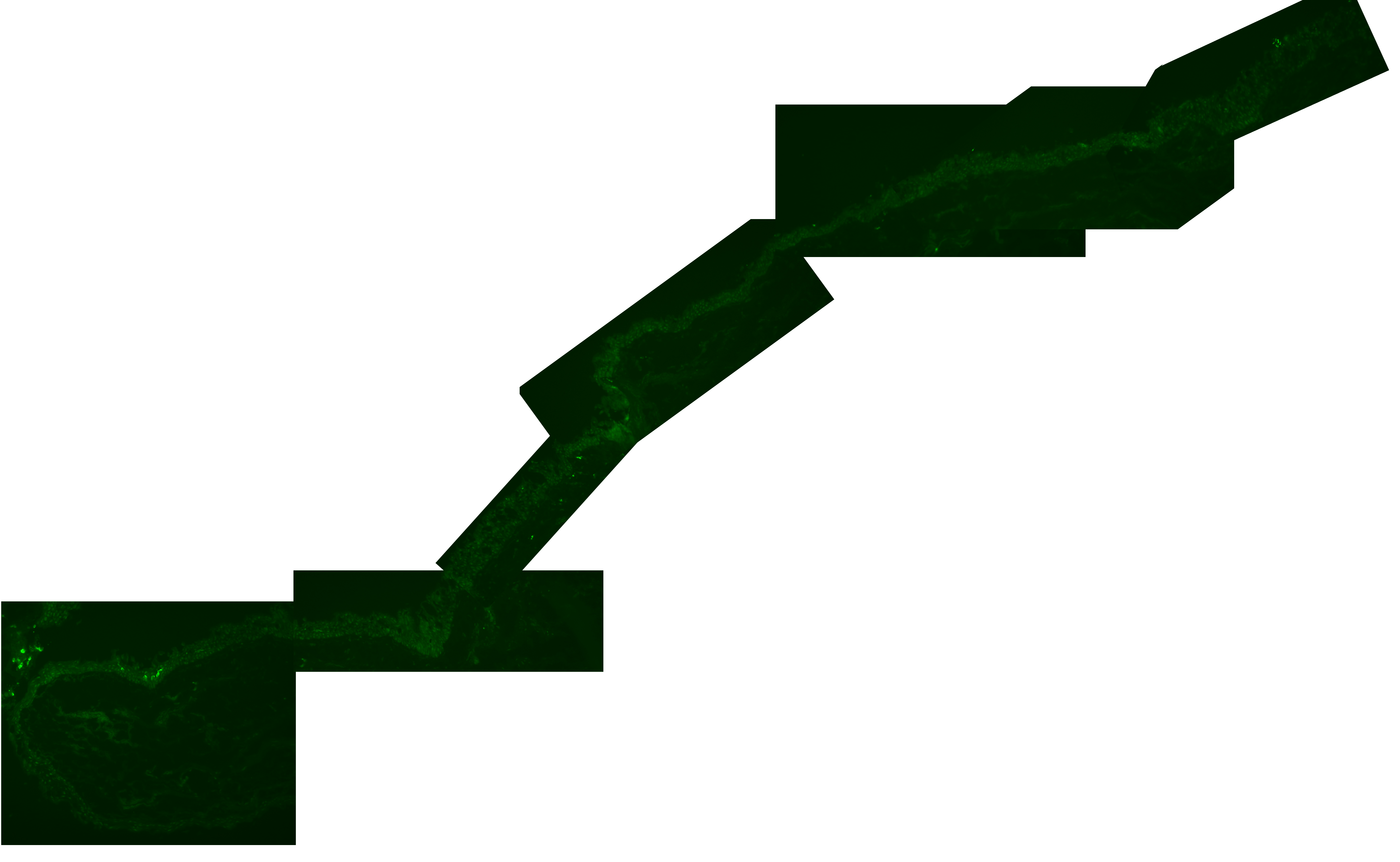

Supplement: Additional File 7 — Primers used for PCR. [file 1755-8794-2-14-S7.tiff]
